# Insights into the design and interpretation of iCLIP experiments

**DOI:** 10.1186/s13059-016-1130-x

**Published:** 2017-01-16

**Authors:** Nejc Haberman, Ina Huppertz, Jan Attig, Julian König, Zhen Wang, Christian Hauer, Matthias W. Hentze, Andreas E. Kulozik, Hervé Le Hir, Tomaž Curk, Christopher R. Sibley, Kathi Zarnack, Jernej Ule

**Affiliations:** 1 Department of Molecular Neuroscience, UCL Institute of Neurology, Queen Square, London, WC1N 3BG UK; 2 The Crick Institute, 1 Midland Road, London, NW1 1AT UK; 3 MRC Laboratory of Molecular Biology, Francis Crick Avenue, Cambridge, CB2 0QH UK; 4 European Molecular Biology Laboratory (EMBL), Meyerhofstrasse 1, 69117 Heidelberg, Germany; 5 Institute of Molecular Biology (IMB), Ackermannweg 4, 55128 Mainz, Germany; 6 Institut de Biologie de l’ENS (IBENS), 46 rue d’Ulm, Paris, F-75005 France; 7 CNRS UMR 8197, Paris Cedex 05, 75230 France; 8 Molecular Medicine Partnership Unit (MMPU), Im Neuenheimer Feld 350, 69120 Heidelberg, Germany; 9 Department of Pediatric Oncology, Hematology and Immunology, University of Heidelberg, Im Neuenheimer Feld 430, 69120 Heidelberg, Germany; 10 Faculty of Computer and Information Science, University of Ljubljana, Tržaška cesta 25, 1000 Ljubljana, Slovenia; 11 Division of Brain Sciences, Department of Medicine, Imperial College London, London, UK; 12 Buchmann Institute for Molecular Life Sciences (BMLS), Goethe University Frankfurt, Max-von-Laue-Str. 15, 60438 Frankfurt am Main, Germany

**Keywords:** Protein–RNA interactions, iCLIP, eCLIP, irCLIP, Binding site assignment, High-throughput sequencing, Polypyrimidine tract binding protein 1 (PTBP1), Eukaryotic initiation factor 4A-III (eIF4A3), Exon-junction complex

## Abstract

**Background:**

Ultraviolet (UV) crosslinking and immunoprecipitation (CLIP) identifies the sites on RNAs that are in direct contact with RNA-binding proteins (RBPs). Several variants of CLIP exist, which require different computational approaches for analysis. This variety of approaches can create challenges for a novice user and can hamper insights from multi-study comparisons. Here, we produce data with multiple variants of CLIP and evaluate the data with various computational methods to better understand their suitability.

**Results:**

We perform experiments for PTBP1 and eIF4A3 using individual-nucleotide resolution CLIP (iCLIP), employing either UV-C or photoactivatable 4-thiouridine (4SU) combined with UV-A crosslinking and compare the results with published data. As previously noted, the positions of complementary DNA (cDNA)-starts depend on cDNA length in several iCLIP experiments and we now find that this is caused by constrained cDNA-ends, which can result from the sequence and structure constraints of RNA fragmentation. These constraints are overcome when fragmentation by RNase I is efficient and when a broad cDNA size range is obtained. Our study also shows that if RNase does not efficiently cut within the binding sites, the original CLIP method is less capable of identifying the longer binding sites of RBPs. In contrast, we show that a broad size range of cDNAs in iCLIP allows the cDNA-starts to efficiently delineate the complete RNA-binding sites.

**Conclusions:**

We demonstrate the advantage of iCLIP and related methods that can amplify cDNAs that truncate at crosslink sites and we show that computational analyses based on cDNAs-starts are appropriate for such methods.

**Electronic supplementary material:**

The online version of this article (doi:10.1186/s13059-016-1130-x) contains supplementary material, which is available to authorized users.

## Background

RNA-binding proteins (RBPs) play crucial roles in all aspects of post-transcriptional gene regulation. To understand the mechanisms of their action, it is essential to identify the endogenous sites of protein–RNA interactions, which has been aided by the development of ultraviolet (UV) crosslinking and immunoprecipitation (CLIP) [1, 2]. During the CLIP protocol, crosslinked protein–RNA complexes are purified and the RNA fragments are released by digesting the protein, resulting in RNAs with a covalently bound peptide at the crosslink site. This is followed by reverse transcription, during which the bound peptide can lead to truncation of complementary DNAs (cDNA) at the crosslink site. The CLIP protocol prepares the cDNA library in a way that requires the reverse transcriptase to read through this peptide, thereby generating only ‘readthrough cDNAs’. Therefore, individual-nucleotide resolution CLIP (iCLIP) was also developed to exploit the ‘truncated cDNAs’ [3]. The cDNA-starts of these truncated cDNAs identify the nucleotide just downstream of the crosslinked peptide. Even though iCLIP amplifies both truncated and readthrough cDNAs, computational comparisons of CLIP and iCLIP cDNAs estimated that over 80% of iCLIP cDNAs truncate at the crosslink sites of most RBPs [4]. Recently, further variants were developed that also amplify truncated cDNAs, including BrdU-CLIP [5], eCLIP [6] and irCLIP [7]. Therefore, understanding the proportion and characteristics of truncated cDNAs in these protocols is essential.

The computational methods that use cDNA-starts to assign RNA-binding sites have been developed along with iCLIP. However, a recent study observed that the starts of long and short iCLIP cDNAs often map to different genomic positions for several RBPs, which leads to non-coinciding cDNA-starts [8]. Here, we focused on experiments produced for polypyrimidine tract binding protein 1 (PTBP1), eukaryotic initiation factor 4A-III (eIF4A3) and the splicing factor U2 auxiliary factor 65 kDa subunit (U2AF2), which represent examples of non-coinciding or coinciding cDNA-starts in introns or exons. eIF4A3 is a component of the exon junction complex (EJC). In vitro biochemical experiments with several splicing substrates demonstrated that the site of EJC deposition is normally expected at nucleotides −20 to −24 upstream of the exon-exon junction (–24..–20 nt) [9]. However, further studies showed that the sequence and structure of a nascent messenger RNA (mRNA) can shift EJC deposition as far as 10 nt away from this expected site [10]. The non-coinciding cDNA-starts in eIF4A3 iCLIP data produced by the previous study were shifted upstream of this expected region and it was proposed that the presence of non-coinciding cDNA-starts might be related to this shift [8]. The study concluded that the use of cDNA-starts may not be appropriate in iCLIP whenever non-coinciding cDNA-starts are prevalent.

To understand if cDNA-starts can be used to assign RNA-binding sites, we further analysed the iCLIP data with high frequency of non-coinciding cDNA-starts. We first examined the position and prevalence of crosslink-induced mutations to confirm previous findings, showing that such mutations are generally >5-fold less common within iCLIP than CLIP cDNAs, regardless of the presence of non-coinciding cDNA-starts [4]. Moreover, we identified RNA motifs that are commonly associated with crosslink sites and found them most highly enriched at cDNA deletions in CLIP, and cDNA-starts in iCLIP, eCLIP and irCLIP, even if non-coinciding cDNA-starts are prevalent. Interestingly, when using the photoactivatable 4-thiouridine (4SU)-based crosslinking in combination with iCLIP, the motifs were more highly enriched at cDNA-starts than at T-to-C transitions. These results demonstrate that the cDNA-starts can reliably be used to determine crosslink sites in iCLIP, regardless of the crosslinking method.

Further analyses demonstrated that presence of sequence and structural constraints at cDNA-ends is the cause of the non-coinciding cDNA-starts. To experimentally validate this finding, we produced additional PTBP1 and eIF4A3 iCLIP experiments, which demonstrate that the prevalence of the non-coinciding cDNA-starts is directly correlated with the extent of cDNA-end constraints. We show that the broad size range of iCLIP cDNAs in these new experiments allows the cDNA-starts to assign binding sites that align with the expected binding motifs (PTBP1) or binding regions (eIF4A3). We conclude that the use of the iCLIP cDNA-starts is appropriate to assign the protein–RNA crosslink sites in iCLIP and related methods.

## Results

### Crosslink sites are identified by cDNA-starts in iCLIP

**Fig. 1:**
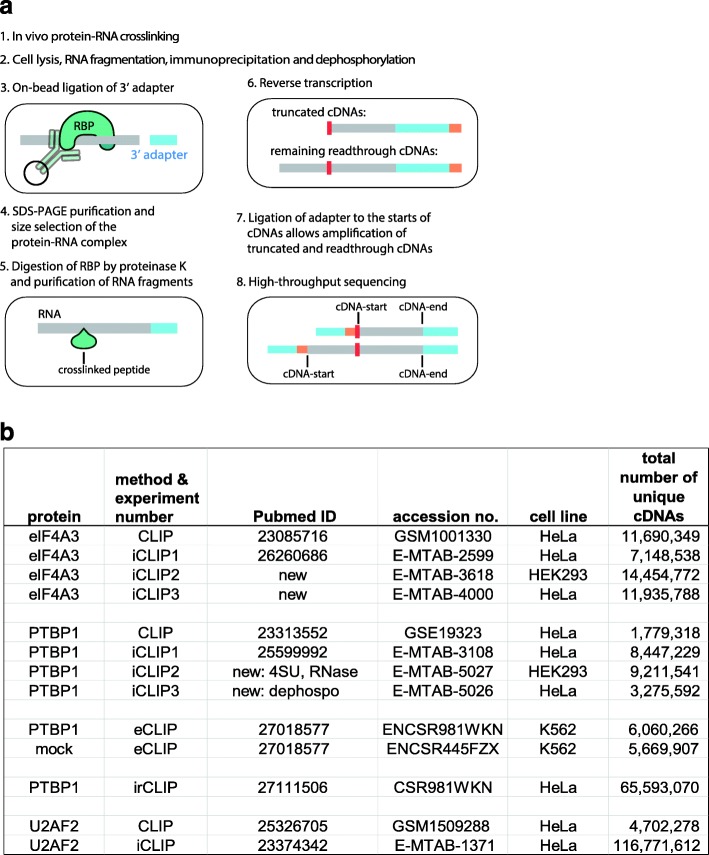
An overview of methods and experiments. **a** A simplified *schematic* of the iCLIP protocol [17]. Before, cells or tissues are irradiated with UV light, which creates covalent bonds between proteins and RNAs that are in direct contact (step 1). After lysis, the crosslinked RNA is fragmented by limited concentration of RNase I and RNA fragments are then co-immunoprecipitated with the RBP (step 2), followed by ligation of a 3′ adapter (step 3). After SDS-PAGE purification (step 4), the crosslinked RBP is removed through proteinase K digestion and purification of RNA fragments (step 5). Reverse transcription is performed with a primer that includes a barcode (orange) containing both an experimental identifier and a unique molecular identifier (UMI) (step 6). The peptide that is on the crosslink site impairs reverse transcription and commonly leads to truncation of cDNAs at the crosslink site. Therefore, two types of cDNAs are generated: truncated cDNAs and readthrough cDNAs. In iCLIP, the cDNA library is prepared in such a way that both truncated and readthrough cDNAs are amplified (step 7). After PCR amplification and sequencing (step 8), both truncated and readthrough cDNAs are present. **b** *Table* summarising the experiments used in this study. *4SU* using 4SU combined with UV-A crosslinking, *RNase* optimised RNase digest conditions including antiRNase inhibitor and increased RNase I concentration, *dephospho* omitting 3′ dephosphorylation

The iCLIP protocol is composed of eight principal experimental steps (Fig. 1a). First, cells or tissues are irradiated with UV light, which can create covalent bonds between an RBP and RNA. Cell lysates are then treated with RNase and the crosslinked RNA fragments are co-immunoprecipitated with the RBP. In the third step, an oligonucleotide adapter is ligated to the 3′ end of RNA fragments. The immunoprecipitated complexes are then separated and visualised by SDS-PAGE and the protein–RNA complex is isolated in a size-specific manner (Additional file 1: Figure S1). The RBP is removed from the RNA through proteinase K digestion, leaving a small peptide at the crosslink site. This impairs the reverse transcription and commonly leads to the truncation of cDNAs at the crosslinked peptide. Hence, iCLIP cDNAs start at the nucleotide just downstream of the crosslinked peptide and they end at the site of RNase cleavage.

To assess how variations in experimental conditions affect the assigned binding sites, we compared published and newly produced experiments for eIF4A3, PTBP1 and U2AF2. For the ease of comparisons, we numerically label the different experiments produced by the same method (Fig. 1b). eIF4A3-iCLIP1 refers to data generated in the previous study [8], while eIF4A3-iCLIP2 and eIF4A3-iCLIP3 were newly produced by the Le Hir and Ule labs, respectively. These are compared to the published eIF4A3 CLIP [11]. The PTBP1-iCLIP1 also refers to data generated in the previous study [12], while PTBP1-iCLIP2 and PTBP1-iCLIP3 were newly produced with deliberate protocol differences. Specifically, 4SU was used to induce crosslinking and RNase I conditions were adjusted in PTBP1-iCLIP2, and the 3′ dephosphorylation step was omitted in PTBP1-iCLIP3. These are compared to the published PTBP1 CLIP [13], eCLIP [6] and irCLIP data [7]. Finally, we also compare the PTBP1 data to U2AF2 CLIP [14] and iCLIP [15].

**Fig. 2:**
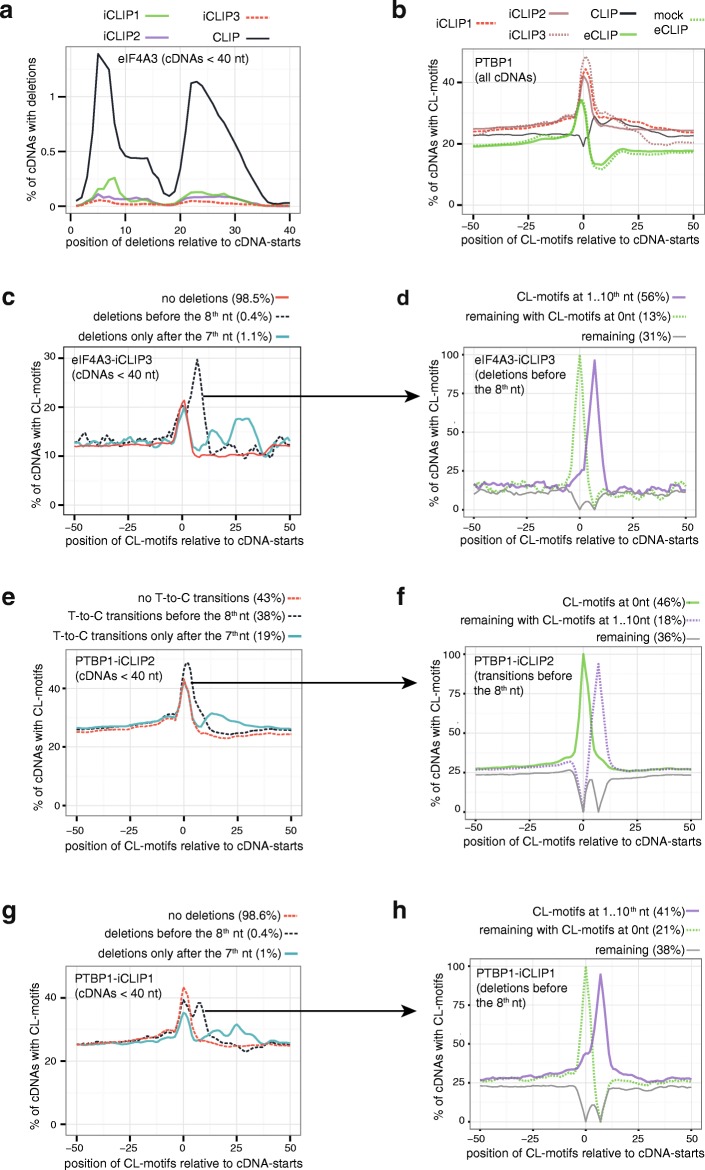
Crosslink-associated (CL)-motifs are enriched at cDNA deletions and cDNA-starts in iCLIP. **a** Proportion of eIF4A3 cDNAs with deletion at each position relative to the cDNA-start. Only cDNAs shorter than 40 nt are examined. **b** Analysis of all PTBP1 experiments examined in this study shows the proportion of cDNAs from each experiment that overlap with a CL-motif at each position relative to the cDNA-start. **c** Proportion of eIF4A3-CLIP3 cDNAs that overlap with a CL-motif at each position relative to the cDNA-start. Only cDNAs shorter than 40 nt are examined; they are divided into those lacking deletions or containing a deletion within the first 7 nt or anywhere in the remaining portion of the cDNA. **d** The cDNAs of eIF4A3-CLIP3 containing a deletion within the first 7 nt are further sub-divided into three categories. First, cDNAs with CL-motifs between the 1st and 10th nucleotide of the cDNA. Second, the remaining cDNAs that contain CL-motifs at the position 0. And third, all remaining cDNAs. The proportion of cDNAs that overlap with a CL-motif at each position relative to the cDNA-start is then plotted for each sub-category. **e** Proportion of PTBP1-iCLIP2 cDNAs that overlap with a CL-motif at each position relative to the cDNA-start. Only cDNAs shorter than 40 nt are examined and are divided into those lacking T-to-C transitions or containing a transition within the first 7 nt or anywhere in the remaining portion of the cDNA. **f** The cDNAs of PTBP1-iCLIP2 containing a T-to-C transition within the first 7 nt are further sub-divided into three categories. First, cDNAs with CL-motifs overlapping the position 0. Second, the remaining cDNAs that contain CL-motifs between the 1st and 10th nucleotide of the cDNA. And third, all remaining cDNAs. Visualisation as in (**d**). **g** Same as (**c**), but for PTBP1-iCLIP1. **h** Same as (**d**), but for PTBP1-iCLIP1

It was proposed that presence of non-coinciding cDNA-starts might indicate that some of these cDNAs have read through the crosslink site during reverse transcription [8]. It has been shown previously that such readthrough cDNAs often contain deletions, which are introduced into cDNAs at the crosslink site during reverse transcription [4, 16]. We compared the proportion of cDNAs with deletions in the different eIF4A3 datasets. Since the rate of sequencing errors rises with increasing cDNA length, we only examined cDNAs shorter than 40 nt for this purpose. Strikingly, a bimodal distribution of deletions is apparent in all datasets, with one peak of deletions close to the cDNA-starts (5..8th nt) and the second close to the cDNA-centres (22..27th nt, Fig. 2a). Thus, the deletions present in iCLIP show the same features as in CLIP and likely inform on the presence of readthrough cDNAs. Importantly, the proportion of deletions is lower by a factor of 5 or more in all eIF4A3 iCLIP experiments compared to CLIP, indicating that readthrough cDNAs represent a minor proportion of iCLIP data.

We used sequence motifs as a second feature that can serve as an identifier of crosslink sites. We defined these sequence motifs based on analysis of eCLIP mock input data that were produced along with the PTBP1 eCLIP [6]. Even though no immunoprecipitation is done, the eCLIP mock data represent RNA fragments crosslinked to RBPs, because the lysate is loaded onto the gel and transferred to a nitrocellulose membrane and the non-crosslinked RNA migrates out of the gel or through the membrane. Thus, eCLIP mock data represent RNAs crosslinked to many different RBPs and should reflect the sequence preferences at crosslink sites that are common to a mixture of RBPs. We identified 10 tetramers that are enriched at cDNA-starts by a factor of 1.5 or more compared to the 10 nt region upstream of the cDNA-starts. Since they serve as a signature of crosslink sites, we refer to them as ‘CL-motifs’ (for UV crosslink-associated motifs). On one hand, these CL-motifs could represent sequence preferences of one or few unknown RBPs that dominate the eCLIP mock input data. On the other hand, all CL-motifs are rich in uridines (see ‘Methods’), which would agree with the hypothesis of preferred UV-C crosslinking to uridines [4]. The CL-motifs are rich in polypyrimidine sequences that are preferentially bound by PTBP1 and U2AF2 [18] and thus it is expected that their enrichment should be especially high at crosslink sites of these proteins. While no further increase in CL-motif enrichment is seen at cDNA-starts of PTBP1-eCLIP, it is reassuring to find their increased enrichment at cDNA-starts of all PTBP1 and U2AF2 iCLIP experiments (Fig. 2b, Additional file 1: Figure S2A).

We also found significant enrichment of CL-motifs at cDNA-starts of all eIF4A3 iCLIP experiments (Fig. 2c, Additional file 1: Figure S2B). eIF4A3 is not thought to bind RNA with sequence specificity based on biochemical and transcriptomic studies [9, 11, 19] and its sequence-independent interaction with RNA is consistent with the properties of DEAD-box proteins [20]. Moreover, we did not find any generic enrichment of CL-motifs at nucleotides −20 to −24 upstream of the exon-exon junctions, where EJC normally binds (data not shown). Thus, it is most likely that CL-motifs only reflect crosslinking preferences in the case of eIF4A3 iCLIP. In contrast to their enrichment at cDNA-starts of all iCLIP experiments, CL-motifs are depleted from the cDNA-starts of all CLIP experiments and instead they are enriched within the sequence of CLIP cDNAs (Fig. 2b, Additional file 1: Figure S2A, B). This agrees with the expected prevalence of truncated cDNAs in iCLIP and readthrough cDNAs in CLIP.

To further assess the validity of CL-motifs, we exploited the bimodal distribution of deletions in the cDNAs shorter than 40 nt, where one peak of deletions is seen in the first 7 nt and a second peak around the centre of cDNAs (Fig. 2a). We separated the cDNAs into three classes: those with deletions in the first 7 nt, those with deletions elsewhere and those with no deletions. If cDNAs contain a deletion in PTBP1 and U2AF2 iCLIP, CL-motifs are most highly enriched at the position of deletion, but not at cDNA-starts, which confirms that they represent readthrough cDNAs (Additional file 1: Figure S2C, D). However, in iCLIP of all three proteins, >90% of cDNAs lack deletions; in these cDNAs, CL-motifs are enriched exclusively at the cDNA-starts, confirming that these largely correspond to truncated cDNAs (Fig. 2c, Additional file 1: Figure S2C, D). In conclusion, analysis of cDNA deletions and CL-motifs indicates that the position of crosslink sites can generally be defined by cDNA-starts in iCLIP.

### cDNA-starts assign crosslink sites in iCLIP regardless of the crosslinking method

We noticed that the cDNAs with deletions in eIF4A3-iCLIP3 contain some CL-motif enrichment at cDNA-starts in addition to the position of deletions (Fig. 2c). To better understand this dual enrichment, we separated those cDNAs with deletion before the 8th nt into three classes (Fig. 2d). Fifty-six percent of cDNAs had the CL-motifs overlapping with the deletion and these had no additional motif enrichment at cDNA-starts. Thirteen percent had CL-motifs at their start, but not at the position of the deletion, and 31% had CL-motifs at neither position. A possible explanation for the dual enrichment is that about 80% of deletions correspond to crosslink sites and about 20% are a result of sequencing errors within truncated rather than readthrough cDNAs. This further indicates that readthrough cDNAs correspond to a minor proportion of iCLIP reads.

Since cDNAs with deletions are rare in iCLIP, we performed a new PTBP1-iCLIP experiment (PTBP1-iCLIP2) in which we incubated cells with 4SU and induced crosslinking with UV-A. We additionally optimised the RNase conditions (see below). In PAR-CLIP, which originally introduced 4SU-mediated crosslinking, the presence of T-to-C transitions indicates the position of crosslink sites [21] and therefore we wished to examine if the same applies to iCLIP when 4SU is used to induce crosslinking. We used CL-motifs to examine the alignment of cDNA-truncations and transitions to crosslink sites. The CL-motifs are CU-rich (see ‘Methods’) and correspond well to the known binding motifs of PTBP1 [18]. Thus, even if 4SU-mediated crosslinking has different sequence preferences, we expect that the CL-motifs should align well to the crosslink sites of PTBP1 due to its binding preferences. The further benefit of using the same CL-motifs for all analyses is that it allows us to directly compare the extent of their enrichment across all different experiments. Notably, we obtained an unexpected misalignment between CL-motifs and transitions in PTBP1-iCLIP2: 57% of cDNAs contained deletions, but CL-motifs were enriched mainly at cDNA-starts, just like in PTBP1-iCLIP1 (compare Fig. 2e and Additional file 1: Figure S2C). In 67% of cDNAs with transitions, the position of the transition mapped to the first few nucleotides close to the cDNA-start. We therefore examined these cDNAs in more detail by dividing them into three classes (Fig. 2f): 46% of these cDNAs contain CL-motifs at the cDNA-start. Eighteen percent contain CL-motifs at the site of transition rather than the cDNA-start. Thirty-six percent contain no CL-motif at either position. A possible explanation for this pattern of enrichment is that about 20% of transitions correspond to crosslink sites and about 75% are a result of other causes. In conclusion, presence of transitions does not separate readthrough and truncated cDNAs in iCLIP, since CL-motifs are equally enriched at cDNA-starts of cDNAs containing or lacking transitions.

While transitions do not overlap well with CL-motifs in PTBP1-iCLIP2, the overlap is better with deletions in PTBP1-iCLIP1. Even though only 1.4% of PTBP1-iCLIP1 cDNAs contain deletions (Fig. 2g), a greater proportion contain CL-motifs at the position of the deletion than at cDNA-starts (Fig. 2h). This indicates that deletions are more reliable than transition to identify crosslink sites in readthrough cDNAs, even when 4SU is used for crosslinking in iCLIP. Taken together, analysis of deletions, transitions and CL-motifs indicates that the incidence of readthrough cDNAs is generally low and that the majority of cDNAs truncate at crosslink sites in iCLIP regardless of the crosslinking method.

### Non-coinciding cDNA-starts result from constrained cDNA-ends

**Fig. 3:**
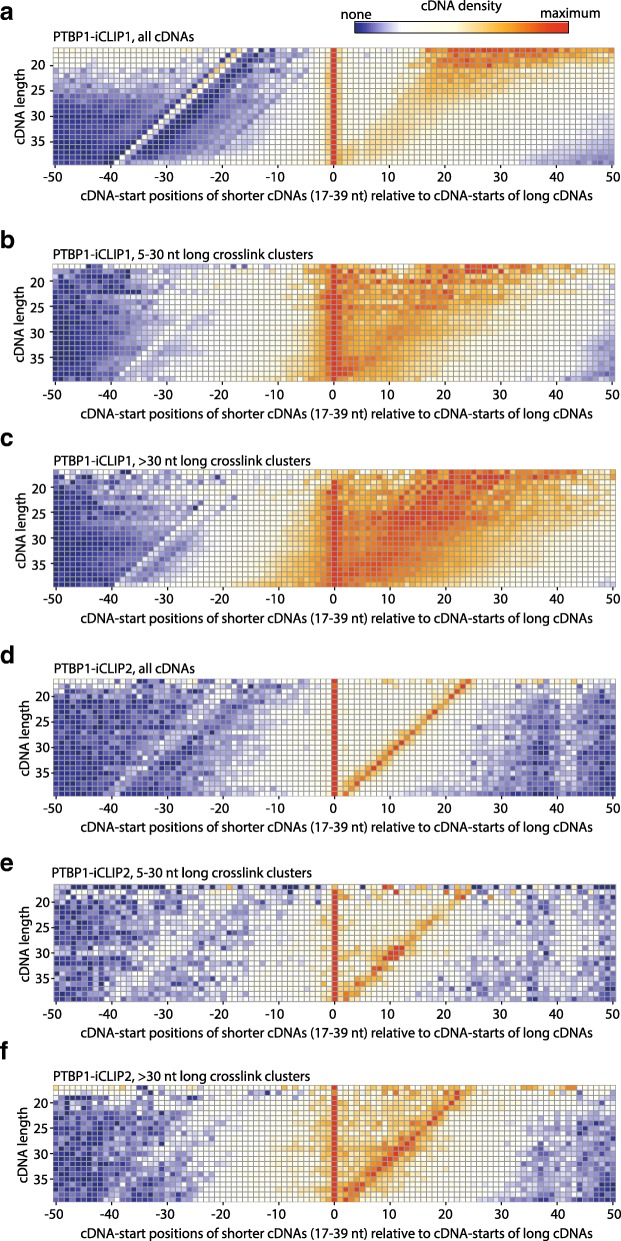
Proportion of non-coinciding cDNA-starts differs between PTBP1 iCLIP experiments. **a** *Heatmap* for PTBP1-iCLIP1 generated using the previously developed software iCLIPro [8] to show the relative positioning of cDNA-starts of shorter iCLIP cDNAs (17–39 nt) compared to cDNA-starts of long cDNAs (longer than 39 nt). **b** As in (**a**), but for cDNAs of PTBP1-iCLIP1 that overlap with 5–30 nt long crosslink clusters. **c** As in (**a**), but for cDNAs of PTBP1-iCLIP1 that overlap with >30 nt long crosslink clusters. **d** As in (**a**), but for PTBP1-iCLIP2. **e** As in (**a**), but for cDNAs of PTBP1-iCLIP2 that overlap with 5–30 nt long crosslink clusters. **f** As in (**a**), but for cDNAs of PTBP1-iCLIP2 that overlap with >30 nt long crosslink clusters

In addition to the model of readthrough cDNAs, the previous study also discussed an alternative model, in which the non-coinciding cDNA-starts could originate from constraints on RNase cleavage, particularly when these are combined with the presence of long binding sites [8]. We examined this alternative model in more detail, since the prevalence of readthrough cDNAs in iCLIP did not appear sufficient to account for the non-coinciding cDNA-starts. We used the tool developed by the previous study (iCLIPro) to examine the prevalence of non-coinciding cDNA-starts in the PTBP1-iCLIP1 dataset. This tool compares the cDNA-start positions of shorter and longer cDNAs and displays overlapping starts by enrichment at position 0, while non-coinciding starts are enriched at other positions. As seen previously [8], we find that the PTBP1-iCLIP1 library contains non-coinciding cDNA-starts (Fig. 3a). To understand if the prevalence of non-coinciding cDNA-starts depends on the length of binding sites, we first identified regions on RNAs where cDNA-starts are significantly clustered, and we refer to these as ‘crosslink clusters’ (see ‘Methods’ for more detail). Notably, the proportion of non-coinciding cDNA-starts increases within crosslink clusters that are longer than 5 nt (Fig. 3b) and this increase is particularly dramatic in clusters longer than 30 nt (Fig. 3c). This analysis reveals that the non-coinciding cDNA-starts originate mainly from long binding sites.

To understand the possible causes and effects of the constrained RNase cleavage, we examined the new PTBP1-iCLIP2 experiment, in which we had also modified the conditions of RNase treatment: in this experiment, we included an inhibitor of endogenous RNases into the lysis buffer (antiRNase which does not inhibit RNase I) and slightly increased the concentration of RNase I compared to PTBP1-iCLIP1. In this way, we hoped to ensure that RNase I, which is not thought to have any sequence specificity, was responsible for fragmenting the RNAs in PTBP1-iCLIP2. Interestingly, the proportion of non-coinciding cDNA-starts is decreased in PTBP1-iCLIP2 (Fig. 3d), and this decrease is particularly apparent when analysing long crosslink clusters (Fig. 3e, f). In addition to the overlapping cDNA-starts, the cDNA-ends in PTBP1-iCLIP2 also often overlap with cDNA-starts (diagonal enrichment in Fig. 3d-f). Both RNase I and UV crosslinking require single-stranded RNA and thus their similar RNA structure preferences are the likely cause for their increased chance of overlap. Taken together, our results show that the prevalence and position of non-coinciding cDNA-starts can vary greatly between different iCLIP experiments performed for the same RBP and thus they are most likely a result of technical differences between these experiments.

**Fig. 4:**
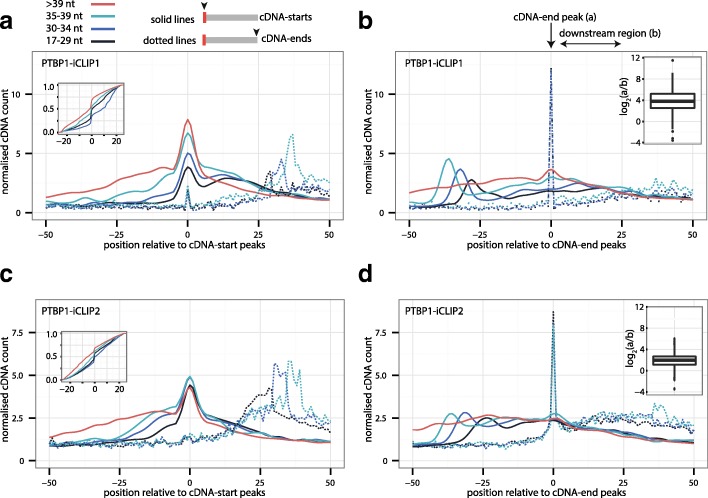
Non-coinciding cDNA-starts are a result of constrained cDNA-ends. **a** The cDNA-starts (*solid lines*) and cDNA-ends (*dotted lines*) of PTBP1-iCLIP1 are plotted around the cDNA-start peak that was identified within each of the 1000 > 30 nt long crosslink clusters that have the highest total cDNA count. cDNAs are divided into four length categories: 17–29 nt, 30–34 nt, 35–39 nt and >39 nt. The *inner plot* shows the empirical cumulative distribution from all four length categories in the region between –25 nt and 25 nt around cDNA-start peaks. **b** As in (**a**), but plotted around the cDNA-end peak that was identified within the 30 nt downstream of each of the 1000 > 30 nt long crosslink clusters that have the highest total cDNA count. The *inner box plot* shows the ratio of cDNA counts (log_2_) at the position 0 (overlapping with cDNA-end peak) compared to the average count of cDNAs in the region from 5 nt to 25 nt downstream of the cDNA-end peak (marked by *horizontal arrow*). **c** As in (**a**), but for PTBP1-iCLIP2. **d** As in (**b**), but for PTBP1-iCLIP2

To understand the technical features that lead to the non-coinciding cDNA-starts in PTBP1-iCLIP1, we examined in more detail the long crosslink clusters in which such cDNAs are most prominent. We restricted all following analyses to the 1000 crosslink clusters with the highest cDNA count to ensure that they have high coverage of diverse cDNA lengths. We identified the position within each crosslink cluster with the highest count of cDNA-starts (cDNA-start peak) and the position downstream of each crosslink cluster with the highest count of cDNA-ends (cDNA-end peak). Next, we categorised cDNAs based on their length and plotted their starts and ends around cDNA-start peaks (Fig. 4a) or cDNA-end peaks (Fig. 4b). As expected for long crosslink clusters, cDNA-starts are broadly distributed around the cDNA-start peaks. We measured the empirical cumulative distribution of cDNA-starts around cDNA-start peaks (Fig. 4a, c – inset), which demonstrates that the distribution of cDNA lengths has a much stronger influence on the position of cDNA-starts in PTBP1-iCLIP1 (Fig. 4a) than PTBP1-iCLIP2 (Fig. 4c). Strikingly, the cDNA-ends of all length categories precisely overlap at the position of cDNA-end peaks in PTBP1-iCLIP1 (Fig. 4b), while they are enriched over a broader region downstream of the cDNA-end peaks in PTBP1-iCLIP2 (Fig. 4d). Indeed, this tight constraint of cDNA-ends in PTBP1-iCLIP1 reveals three distinct peaks of cDNA-starts for each category of cDNA lengths (Fig. 4b), while these peaks are less prominent in the PTBP1-iCLIP2 library, in which the fold change for cDNA-end constraint is decreased by half (Fig. 4b, d – inner box plot). We conclude that the presence of non-coinciding cDNA-starts is reduced in PTBP1-iCLIP2 due to the decreased constraints at cDNA-ends.

### PTBP1 binding sites can be assigned correctly despite non-coinciding cDNA-starts

Analysis of the PTBP1-iCLIP1 demonstrated that the non-coinciding cDNA-starts are most enriched within long crosslink clusters. Thus, we speculated that analysis of long binding sites might detect non-coinciding cDNA-starts also in those iCLIP datasets previously analysed by the iCLIPro tool, even if they had not been detected by iCLIPro. For example, U2AF2-iCLIP appears to lack non-coinciding cDNA-starts when analysed by iCLIPro [8] and so we looked at iCLIP data for this protein in more detail.

**Fig. 5:**
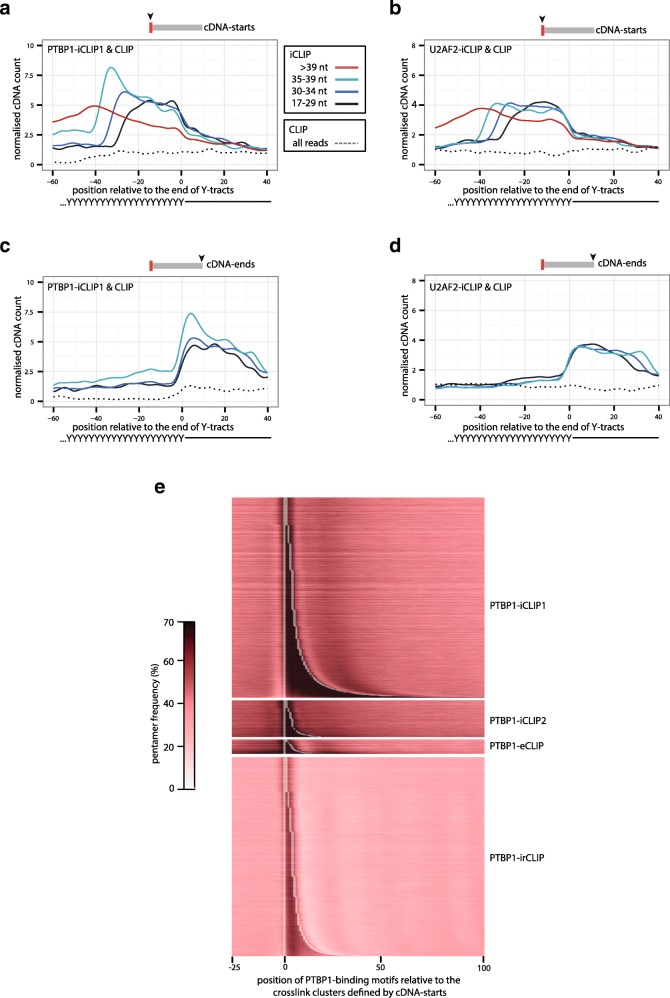
Non-coinciding cDNA-starts are required to map the crosslink sites within Y-tracts. **a** The cDNA-starts of PTBP1-iCLIP1 and CLIP experiments are plotted around the ends of >35 nt Y-tracts that are annotated as T-rich or TC-rich low-complexity sequence in the human genome (hg19). cDNAs of PTBP1-iCLIP1 are divided into four length categories: 17–29 nt, 30–34 nt, 35–39 nt and >39 nt**. b** Same as (**a**), but using U2AF2-iCLIP and CLIP cDNAs. **c** Same as (**a**), but showing the positions of cDNA-ends. **d** Same as (**b**), but showing the positions of cDNA-ends. **e** *Heatmap* showing the coverage of PTBP1-binding motifs at the PTBP1-iCLIP1, PTBP1-iCLIP2, PTBP1-eCLIP or PTBP1-irCLIP crosslink clusters that were defined with a 3-nt clustering window. Each *row* shows the average coverage for 300 clusters of similar length, sorted from shortest to longest clusters. The *white line* marks the nucleotide preceding the start and the nucleotide following the median end of all clusters that were combined in each row. A *colour key* for the coverage per nucleotide of the PTBP1-binding motifs is shown on the *right*

Both PTBP1 and U2AF2 preferentially bind to pyrimidine tracts (Y-tracts) [14, 15, 18] and therefore we defined the coordinates of potential PTBP1 and U2AF2 binding sites independently of iCLIP data. Specifically, we compared the crosslinking of these two proteins across the longest computationally identified Y-tracts that are annotated in the human genome as T-rich or TC-rich ‘low complexity sequences’ and are located mainly at deep intronic positions. Interestingly, PTBP1-iCLIP1 and U2AF2-iCLIP have a similar presence of non-coinciding cDNA-starts within these long Y-tracts. The short iCLIP cDNAs identify the crosslink sites close to the 3′ region of the Y-tracts, while longer cDNAs identify crosslink sites that are located further towards the 5′ region (Fig. 5a, b). These non-coinciding cDNA-starts of all cDNA length categories correctly identify crosslink sites, because they are enriched almost exclusively within the Y-tracts, which these proteins are known to bind (Additional file 1: Figure S3).

Notably, the cDNA-ends are constrained to positions downstream of the Y-tracts both in PTBP1-iCLIP1 and U2AF2-iCLIP (Fig. 5c, d). Since cDNA-ends represent the sites of RNase cleavage, this most likely indicates inefficient RNase cleavage within the Y-tracts. Thus, the presence of non-coinciding cDNA-starts reflects constrained positions of cDNA-ends. In this context, the broad size range of iCLIP cDNAs is crucial to overcome the constraints at cDNA-ends, thereby enabling the non-coinciding cDNA-starts to detect crosslink events across the long Y-tracts. Moreover, the long cDNAs are particularly important, since they can truncate at crosslink sites that are located far from the site of RNase cleavage. In doing so, they identify crosslink sites across the entire length of the long binding sites.

Our analysis of Y-tracts indicates that non-coinciding cDNA-starts do not necessarily have a negative effect on the assignment of binding sites. To examine this notion more comprehensively, we compared the sequence features of crosslink clusters defined by PTBP1-iCLIP1 and PTBP1-iCLIP2. First, we identified PTBP1-binding motifs by searching for pentamers that are most highly enriched around the cDNA-starts in PTBP1-iCLIP2 (see ‘Methods’). Then, we visualised the position of these PTBP1-binding motifs around the crosslink clusters that were identified by cDNA-starts in the different iCLIP, eCLIP or irCLIP experiments (Fig. 5e). This confirmed that enrichment of the PTBP1-binding motifs correctly overlaps with the starts and ends of crosslink clusters regardless of which crosslinking type or which variant of library preparation protocol was used. Moreover, the high prevalence of non-coinciding cDNA-starts in PTBP1-iCLIP1 does not affect the high resolution of the method. Taken together, we conclude that the use of cDNA-starts is appropriate for the computational analysis of data produced by iCLIP or any related method that is capable of efficiently amplifying truncated cDNAs.

### Efficient RNase I-mediated RNA fragmentation minimises the cDNA-end constraints

**Fig. 6:**
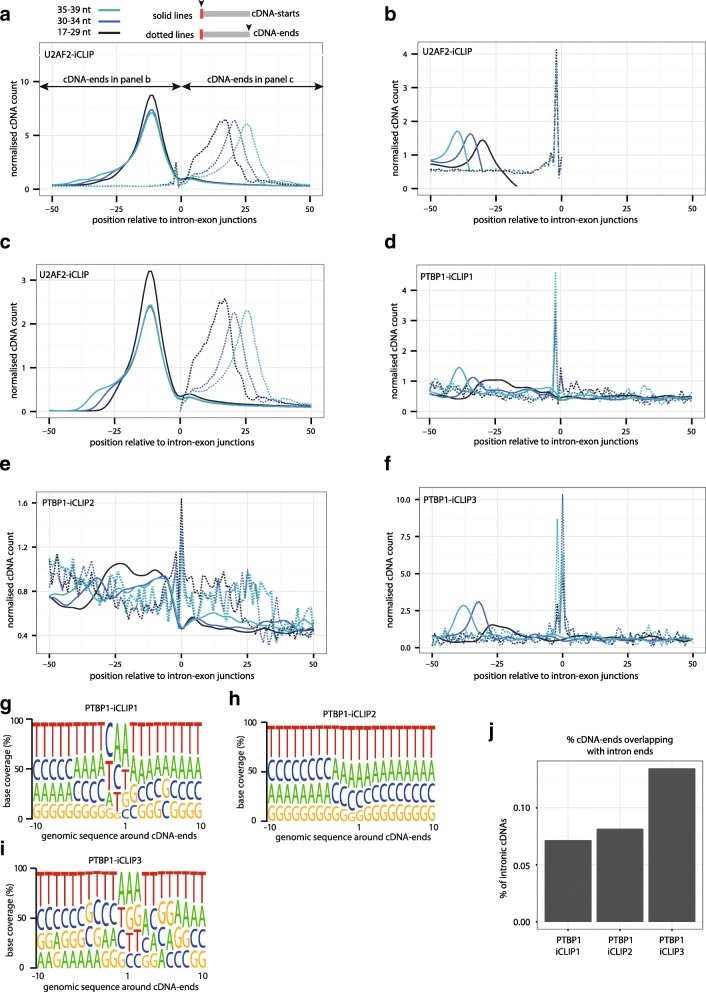
Constrained cDNA-ends affect the cDNA-starts at 3′ splice sites. **a** The cDNA-starts (*solid lines*) and cDNA-ends (*dotted lines*) of U2AF2-iCLIP are plotted around intron-exon junctions (position 0 being the first nucleotide of the exon). cDNAs are divided into three length categories: 17–29 nt, 30–34 nt and 35-39 nt; the distribution of all cDNAs together is shown in *grey*. **b** Same as (**a**), but using only cDNAs that end in the intron. **c** Same as (**a**), but using only cDNAs that end in the exon. **d** Same as (**a**), but showing PTBP1-iCLIP1 cDNA-starts (*full lines*) and cDNA-ends (*dotted lines*). **e** Same as (**a**), but showing PTBP1-iCLIP2 (using 4SU and optimised RNase conditions) cDNA-starts (*full lines*) and cDNA-ends (*dotted lines*). **f** Same as (**a**), but showing PTBP1-iCLIP3 (omitting 3′ dephosphorylation) cDNA-starts (*full lines*) and cDNA-ends (*dotted lines*). **g** The composition of genomic nucleotides around iCLIP cDNA-ends from PTBP1-iCLIP1. **h** Same as (**g**), but showing PTBP1-iCLIP2. **i** Same as (**g**), but showing PTBP1-iCLIP3. **j** Proportions of cDNAs that map to introns which contain cDNA-ends at positions overlapping the last two nucleotides of introns. PTBP1-iCLIP1 and PTBP1-iCLIP2 are compared to PTBP1-iCLIP3 iCLIP, which was performed without using PNK to dephosphorylate RNAs in step 2. This enriches for RNAs that contain a 3′ OH, which can occur when they are cleaved at their 3′ end independently of RNase I, such as the 3′ ends of intron lariats

The cDNA-end corresponds to the position where the RNA fragment was cleaved by the RNase (Fig. 1a, step 2). As described earlier, we optimised the conditions of RNase treatment in the PTBP1-iCLIP2 experiment to ensure that RNase I is the primary cause of RNA fragmentation. This indicates that RNA fragmentation by other factors might have caused the high cDNA-end constraints in PTBP1-iCLIP1. To investigate this possibility, we first assessed cDNA positions at the 3′ splice sites, since these are subject to endogenous RNA cleavage by the spliceosome. While PTBP1 binds to Y-tracts at specific 3′ splice sites to repress alternative splicing, U2AF2 binds to most 3′ splice sites [14, 15, 18]. Interestingly, a peak of cDNA-ends is present at the last intronic nucleotide, even though most cDNA-ends are in the exonic sequence (Fig. 6a). The U2AF2-iCLIP cDNAs of all length categories that end in terminal part of introns have non-coinciding cDNA-starts (Fig. 6b), while the cDNAs that end in the exon have fully coinciding cDNA-starts (Fig. 6c).

The intronic U2AF2-iCLIP cDNA-ends are constrained to the position where the 3′ splice site is cleaved by the endogenous spliceosome. However, the cDNA-ends in exons are not constrained, most likely because they result from cleavage of pre-mRNA by RNase I. To test this hypothesis, we exploited the fact that intron lariats lack a phosphate at their 3′ end and thus no 3′ dephosphorylation is needed at step 2 of the protocol to detect them in iCLIP (Fig. 1a). We therefore produced another PTBP1 iCLIP experiment (PTBP1-iCLIP3), in which we omitted dephosphorylation from step 2 and continued directly to ligation of the 3′ adapter in step 3 (Fig. 1a). Since RNA fragments cleaved at their 3′ end by RNase I contain a 3′ phosphate, they were not ligated to the 3′ adapter (Fig. 1a, step 3) and therefore only those RNA fragments cleaved by other means were amplified in PTBP1-iCLIP3. Notably, both in PTBP1-iCLIP1 and PTBP1-iCLIP3, the cDNA-ends at 3′ splice sites are strongly constrained to the end of introns, while this constraint is minor in PTBP1-iCLIP2 (Fig. 6d–f). Thus, non-coinciding cDNA-starts predominate at 3′ splice sites in PTBP1-iCLIP1 and PTBP1-iCLIP3, while in PTBP1-iCLIP2 most cDNA-starts coincide in the region of 20 nt to 5 nt upstream of the intron-exon junction. This suggests that the RNAs overlapping with the 3′ splice sites were fragmented by spliceosome-mediated cleavage in PTBP1-iCLIP1 and PTBP1-iCLIP3 and by RNase I in PTBP1-iCLIP2 and in U2AF2-iCLIP. It is this difference that appears to explain the variation in the prevalence of non-coinciding cDNA-starts at 3′ splice sites.

To further compare the characteristics at cDNA-ends, we visualised the nucleotide composition of cDNA-ends for the three PTBP1 iCLIP experiments. In PTBP1-iCLIP2, for which we used the increased RNase I concentration, we observed almost no sequence biases at cDNA-ends, in agreement with the lack of sequence specificity of RNase I (Fig. 6h). In contrast, a preference for adenosines was observed at the cDNA-ends in PTBP1-iCLIP1 and PTBP1-iCLIP3, suggesting that this preference results from an RNase I-independent fragmentation of RNAs (Fig. 6g–i). Spliceosome-mediated RNA cleavage contributes to only about 0.1% of these fragments (Fig. 6j) and therefore the primary cause of RNase I-independent fragmentation remains to be identified. Nevertheless, we can clearly conclude that the efficient use of RNase I avoids the constraints at cDNA-ends in iCLIP and this decreases the incidence of non-coinciding cDNA-starts.

### Sequence or structure preferences of RNA fragmentation can constrain the cDNA-ends

**Fig. 7:**
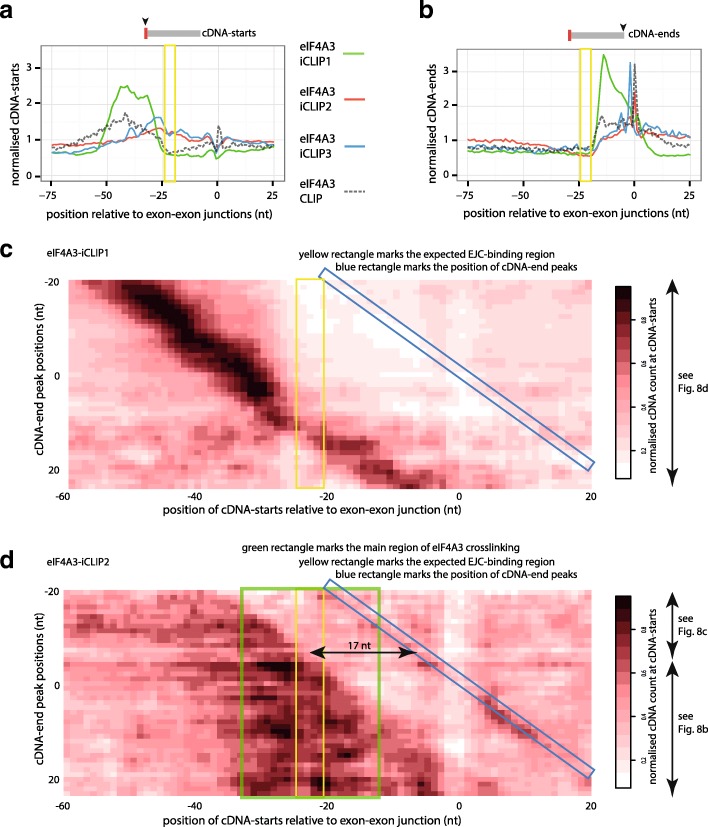
A broad cDNA length range ameliorates the effects of constrained cDNA-ends. **a** The cDNA-starts of eIF4A3 iCLIP and CLIP experiments are plotted around the 1000 exon-exon junctions with the highest number of cDNAs. **b** Same as (**a**), but showing cDNA-ends. **c** *Heatmap* showing the position of cDNA-starts in eIF4A3-iCLIP1 around the 1000 exon-exon junctions with the highest number of cDNAs. Junctions are sorted according to their cDNA-end peak position. Each *row* shows the average of cDNA counts at all junctions with a cDNA-end peak at the indicated position. The values are normalised against the maximum value across all rows. On the *right*, the *arrows* mark parts of the figure in which binding site assignment corresponds to the *schematic* shown in Fig. 8d. *Coloured rectangles* mark the main region of eIF4A3 crosslinking (*green*), the expected EJC-binding region (*yellow*) and the position of the cDNA-end peak (*blue*). **d** Same as (**c**), but for eIF4A3-iCLIP2. The *arrow* in the figure marks the 17 nt minimal distance between cDNA-starts and the expected EJC-binding region that is required for cDNA-starts to be able to identify crosslink sites within the binding site. On the *right*, the *arrows* mark sections that correspond to the schematics shown in Fig. 8c, b

Both U2AF2 and PTBP1 primarily bind to pre-mRNAs and therefore we also wished to assess the impact that constraints at cDNA-ends may have on RBPs binding mature mRNAs. For this purpose, we examined the iCLIP and CLIP cDNA libraries produced for eIF4A3. The position of cDNA-starts varies greatly between different eIF4A3 experiments (Fig. 7a). As observed by the previous study, the cDNA-starts in eIF4A3-iCLIP1 are shifted to positions upstream of the expected EJC-binding region (yellow rectangle in Fig. 7a) [8]. In contrast, the cDNA-starts of eIF4A3-iCLIP2 and eIF4A3-iCLIP3 overlap with the expected binding region. The cDNA-starts of eIF4A3-CLIP are shifted upstream of eIF4A3-iCLIP2 and eIF4A3-iCLIP3, which agrees with the likely prevalence of truncated cDNAs in iCLIP and readthrough cDNAs in CLIP.

Next, we asked how the position of cDNA-ends may influence the position of cDNA-starts. For this purpose, we first examined the positions of cDNA-ends by summarising them across all exon-exon junctions. The cDNA-ends in eIF4A3-iCLIP1 are highly enriched in a region immediately downstream of the expected EJC-binding region (mainly positions –18..0 nt relative to the junctions), but they are also more broadly distributed further downstream of the expected EJC-binding region, including in the downstream exon (Fig. 7b). To understand why the positions of cDNA-ends are so different in eIF4A3-iCLIP1 compared to the remaining experiments, we first identified the cDNA-end peak, corresponding to the position with the highest count of cDNA-ends at each junction. We then grouped all exon-exon junctions that had the same position of the cDNA-end peak relative to the junction. Both in eIF4A3-iCLIP1 and eIF4A3-iCLIP2, each junction has a preferred position of cDNA-ends, indicating that both experiments show a strong cDNA-end constraint at individual junctions (marked by blue rectangle in Additional file 1: Figure S4A, B).

To further understand the causes for different cDNA-end positions in eIF4A3-iCLIP1, we assessed the RNA sequence and structure preference at cDNA-ends. The cDNA-end peak in eIF4A3-iCLIP2, but not eIF4A3-iCLIP1, coincides with a strong decrease in pairing probability (Additional file 1: Figure S4C, D). Since most endonucleases cut at single-stranded RNA, this indicates that the RNA fragments were cut at their 3′ end by an endonuclease in eIF4A3-iCLIP2, while additional factors may contribute to the RNA fragmentation in eIF4A3-iCLIP1. In eIF2A3-iCLIP2, but not eIF4A3-iCLIP1, we also observe a second peak of cDNA-ends precisely at the end of the exon (position –1) (Fig. 7b, Additional file 1: Figure S4B). This probably reflects the deposition of eIF4A3 on the spliced exon intermediate, as would be expected based on previous biochemical studies [22–24]. The nucleotide composition at cDNA-ends also differs between eIF4A3-iCLIP1 and eIF4A3-iCLIP2 (Additional file 1: Figure S4E, F). These differences suggest that RNA was fragmented by different mechanisms in eIF4A3-iCLIP1 and eIF4A3-iCLIP2, but this remains to be fully understood. Both eIF4A3-iCLIP2 and eIF4A3-iCLIP3 have a strong enrichment of adenosine at the position following the cDNA-end peak, indicating a potential for RNase I-independent fragmentation (Additional file 1: Figure S4G). However, the published eIF4A3-CLIP data used RNase T1 to fragment the RNA [11], which prefers to cut after the guanosine, in agreement with a guanosine enrichment at the position preceding the cDNA-end peaks (Additional file 1: Figure S4H). Taken together, these findings indicate that differences in RNA fragmentation conditions can dramatically affect the structural and sequence features at cDNA-ends in CLIP and iCLIP experiments and thus they can constrain the positions of cDNA-ends in multiple different ways. The impact of these constraints becomes clear when aligning the junctions to the position of cDNA-end peak, which demonstrates that the position of each length category of cDNAs is defined by the position of cDNA-ends (Additional file 1: Figure S4I–K).

To understand the constraints at cDNA-ends at the level of individual exon-exon junctions, we examined the exon-exon junctions with highest coverage of cDNAs in greater detail. For this purpose, we focused on the 1000 junctions with the highest cDNA count. This demonstrates that the cDNA-ends are largely restricted to a single position in the eIF4A3-iCLIP3 experiment, while they are more variable in eIF4A3-iCLIP1. As a result, the cDNA-starts often coincide in eIF4A3-iCLIP1, but are fully non-coinciding in eIF4A3-iCLIP3 (Additional file 1: Figure S5). This again demonstrates that the cDNA-end constraints are the primary cause of non-coinciding cDNA-starts in iCLIP. These constraints therefore need to be considered when interpreting the position of binding sites assigned by iCLIP and related methods.

### A broad range of cDNA lengths compensates for the constrained cDNA-ends

To understand how the constraints at cDNA-ends influence the positions of cDNA-starts, we grouped all exon-exon junctions that had the same position of the cDNA-end peak and visualised the position of cDNA-starts (Fig. 7c, d). This confirms that the position of cDNA-ends (marked with blue rectangle) has a strong effect on the position of the identified crosslink sites. Notably, this effect is a lot more pronounced for eIF4A3-iCLIP1, because cDNA-starts are enriched within a narrowly defined distance from the cDNA-ends (Fig. 7c). Analysis of the cDNA length distribution of the examined experiments shows that eIF4A3-iCLIP1 has the largest proportion of cDNAs that are shorter than 39 nt (58%) and most of these cDNAs are in the range of 27–38 nt long (Additional file 1: Figure S6). This indicates that a narrow range of cDNA lengths dominates the eIF4A3-iCLIP1 library and this range of cDNA lengths defines the distance at which cDNA-starts are positioned relative to cDNA-ends. For comparison, only 36% of cDNAs are shorter than 39 nt in eIF4A3-iCLIP3 and the size distribution is more even in eIF4A3-iCLIP2 (Additional file 1: Figure S6A). As a result of the narrow range of cDNA-starts, the cDNA-starts in eIF4A3-iCLIP3 rarely identify crosslink sites within the expected EJC-binding region (marked by the yellow rectangle).

In eIF4A3-iCLIP2, cDNAs have a broad range of lengths, which allows to identify crosslink positions over a broad area upstream of the cDNA-end peak, including the expected EJC-binding region (Fig. 7d). Nevertheless, eIF4A3-iCLIP2 does not identify crosslinking within the expected EJC-binding region at a subset of exon-exon junctions; at these junctions, the cDNA-ends are positioned closer than 17 nt to this region (top portion of the heatmap in Fig. 7d). Since only cDNAs longer than 16 nt are normally isolated by the iCLIP procedure and short cDNAs rarely map to a unique genomic position, it would be very challenging to identify crosslink sites closer than 17 nt to cDNA-ends. This demonstrates that to comprehensively identify crosslink sites within the binding region, the cDNA-ends should ideally be at least 17 nt away from the binding region.

In eIF4A3-iCLIP2, the large majority of cDNA-ends are present more than 17 nt downstream of the expected EJC-binding region (Fig. 7b). This decreased constraint on cDNA-ends and the broad range of cDNA lengths are the most likely reasons for the capacity of eIF4A3-iCLIP2 to identify crosslink sites over the EJC-binding region at most exon-exon junctions. Indeed, most crosslinking in eIF4A3-iCLIP2 is seen within the expected EJC-binding region, as well as approximately 10 nt on each side of this region (marked with green rectangle in Fig. 7d). In conclusion, the broad range of cDNA lengths can overcome the cDNA-end constraints by producing the non-coinciding cDNA-starts that can more comprehensively identify crosslink sites.

## Discussion

**Fig. 8:**
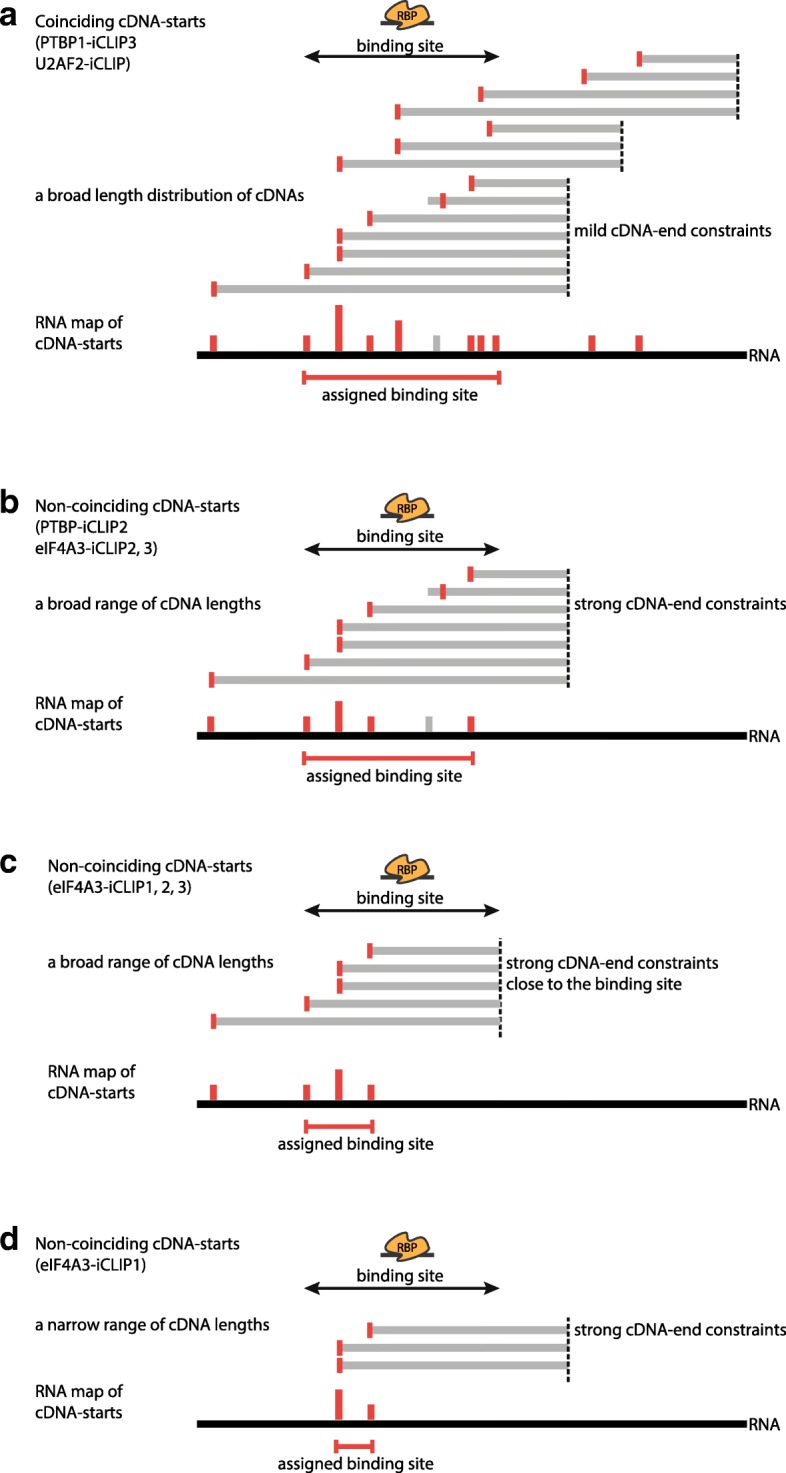
A *schematic* explaining how different extents of cDNA-end constraints affect binding site assignment. **a** If the iCLIP library contains a broad range of cDNA lengths and unconstrained positions of cDNA-ends, then crosslink sites are identified in an unbiased manner, allowing assignment of the full binding site (RNA map at the bottom). The crosslink sites assigned by cDNA-starts are marked in *red bars* and a *grey bar* marks a crosslink site that is incorrectly assigned by a readthrough cDNA. **b** If cDNA-ends are constrained, most likely as a result of biased RNase cleavage, then the resulting cDNA-starts do not coincide. Nevertheless, if a broad distribution of cDNA lengths is available and the cDNA-ends are placed far enough from the binding site, then crosslink sites can still be identified across the full binding site, allowing correct assignment, as was seen in the case of eIF4A3-iCLIP2 (Fig. 7d). **c** If cDNA-ends are constrained to a position very close to the binding site, then those cDNAs that truncate at crosslink sites in the 3′ region of the binding site are too short to be isolated and mapped to the genome. Therefore, crosslink sites are identified only in the 5′ region of the binding site, leading to an overly narrow assignment of binding sites, as was seen in some of the sites identified by eIF4A3-iCLIP1 and eIF4A3-iCLIP2 (Fig. 7c, d). **d** If cDNA-ends are constrained and an iCLIP library contains a narrow distribution of cDNA sizes, then cDNA-end constraints lead to an overly narrow assignment of binding regions, as was seen in the case of eIF4A3-iCLIP1 (Fig. 7c)

Our study demonstrates that use of cDNA-starts is appropriate to assign protein–RNA crosslink sites with iCLIP and related methods, regardless of the crosslinking method. Our findings also underscore the importance of fully optimising the iCLIP conditions with the goal to produce a broad range of cDNA lengths with a minimal cDNA-end constraint. We find that crosslink sites are assigned by cDNA-starts even if non-coinciding cDNA-starts are present, since these are a result of cDNA-end constraints, which can have diverse causes (Fig. 8). The constrained cDNA-ends become problematic when they are present close to the binding site (Fig. 8c) or when a narrow range of cDNA lengths dominates the library (Fig. 8d). In these cases, only a portion of the binding site might be assigned and this portion is likely to locate at the upstream region of binding sites (Fig. 8c, d). In contrast, a broad range of cDNA lengths can compensate for cDNA-end constraints and in this case the presence of non-coinciding cDNA-starts does not detrimentally influence binding site assignment (Fig. 8b).

A previous study suggested that the non-coinciding cDNA-starts might reflect a prevalence of readthrough cDNAs [8]. Here, we show four independent approaches to examine non-coinciding cDNA-starts in PTBP1 and eIF4A3 iCLIP, all of which lead us to conclude that non-coinciding cDNA-starts are unrelated to readthrough cDNAs. First, we show that CL-motifs are enriched mainly at cDNA-starts in iCLIP, but not in CLIP. This also applies to the PTBP1-iCLIP2 experiment in which 4SU was used for crosslinking. Second, we find a much lower proportion of crosslink-induced deletions in eIF4A3 iCLIP compared to CLIP data, as was also observed previously for other RBPs [4]. Moreover, even though the proportion of transitions is high in the PTBP1-iCLIP2, analysis of CL-motifs indicates that a minority of transitions correspond to crosslink sites, while most crosslink sites overlap with cDNA truncations. This agrees with the enrichment of binding motifs at cDNA-starts in CPSF30 iCLIP, where 4SU was also used for crosslinking [25]. This conclusion is specific for iCLIP, since transitions can precisely assign the crosslink site in PAR-CLIP [21], because only readthrough cDNAs are amplified and usually only one transition is present in the sequenced read. Third, we show that the non-coinciding cDNA-starts in iCLIP result from the constrained cDNA-ends and that their prevalence is greatly diminished when RNase I is the primary source of RNA fragmentation. Fourth, while cDNA-starts of readthrough cDNAs could lead to spurious assignment of crosslink sites upstream of the expected binding regions, we find that the binding sites are correctly assigned by PTBP1-iCLIP1 as well as by eIF4A3-iCLIP2 and eIF4A3-iCLIP3. Thus, we find that prevalence of non-coinciding cDNA-starts is unrelated to the presence of readthrough cDNAs.

The presence of non-coinciding cDNA-starts previously served as an argument for using cDNA-centres instead of cDNA-starts because the cDNA-starts are shifted to the region upstream of the expected binding sites in eIF4A3-iCLIP1 [8]. However, we now find that other eIF4A3 iCLIP experiments also contain non-coinciding cDNA-starts, but their cDNA-starts correctly identify crosslink sites; this indicates that the non-coinciding cDNA-starts are not the cause of shifted binding site assignment in eIF4A3-iCLIP1. We now show that this shift is caused by the presence of cDNA-ends just downstream of binding sites, which is unique to eIF4A3-iCLIP1. We also show that the non-coinciding cDNA-starts are an indirect signature of sequence and structure biases at cDNA-ends, which reflect RNase preferences. It has recently been shown that the sequence bias of RNases can be incorporated into models that predict protein-RNA binding [26]. It remains unclear what causes the unusually high constraints at cDNA-ends in some of the experiments. Multiple sources of RNA fragmentation could lead to such preferences, including the cleavage of intron-exon boundaries upon splicing, specificity of RNA cleavage by exogenous or endogenous RNases, RNA fragmentation during sonication or spontaneous hydrolysis. Non-specific RNases, such as RNase I, should be used instead of the sequence-specific RNases, such as the RNase A, T1 or micrococcal nuclease. Moreover, as we demonstrate with the PTBP1-iCLIP2 experiment, it is important that cleavage by RNase I is the dominant source of RNA fragments. The optimal RNase conditions can be confirmed by visualisation of protein-RNA complexes after their separation with SDS-PAGE, as in the published guidelines [3, 17, 27].

We also show that additional aspects of the iCLIP protocol need careful optimisation to avoid cDNA-end constraints. The 3′ dephosphorylation of RNA fragments needs to be efficient (Fig. 1, step 2), since this is necessary for efficient 3′ adapter ligation to the RNA 3′ ends produced by RNase I (Fig. 1, step 3). While previous studies found sequence and structural biases in the RNA ligase-mediated 3′ adapter ligation [28, 29], we show that PTBP1-iCLIP2 cDNA-ends do not have much sequence bias, indicating that RNA ligation is not the reason for the constraints in the other experiments. However, it is important that the ligation is efficient, so that ideally most RNA fragments become ligated to the 3′ adapter, which minimises potential biases. Finally, purification of cDNAs should be performed in a way that maintains a broad range of cDNA lengths in the final amplified library. This should ideally include isolation of both short and long cDNAs to maximise mapping of crosslink sites that are located either close or far from the site of RNase cleavage, respectively. Moreover, it indicates that special procedures for genomic mapping of short cDNAs may be beneficial; for example, due to the problem that short reads often map at multiple genomic positions, mapping of short cDNAs could be narrowed down to the genomic regions where longer cDNAs map. In sum, it is important to ensure that RNase I is the primary source of RNA fragmentation, that 3′ dephosphorylation of RNA fragments is efficient and that the cDNA library has a broad range of cDNA sizes.

We show that use of cDNA-starts is appropriate to assign protein–RNA crosslink sites with iCLIP. Interestingly, we find that the number of assigned crosslink clusters can vary greatly between the different datasets in a manner that does not necessarily correlate with the number of unique cDNAs that are present in the library (Figs. 1b, 5e). These differences might reflect variable amounts of co-purified non-specific RNAs in the different experiments, which could result from the use of different antibodies and purification conditions. To draw more solid conclusions, direct comparisons between multiple methods will need to be done for a larger number of diverse RBPs.

It is clear, however, that identification of long binding sites can be particularly challenging. Presence of long cDNAs is required to identify crosslink sites across the complete length of long PTBP1 binding sites. Moreover, RNase cleavage sites need to be far enough from the EJC-binding site in order to identify contacts within 10 nt on either side of the expected EJC-binding region by the eIF4A3 iCLIP. This is compatible with the previous findings that the precise position of EJC binding can vary between different junctions, which can be influenced by RNA sequence and structure, or by other RBPs that bind in the vicinity [10, 11, 19]. Moreover, crosslink sites positioned further from the expected binding site might reflect eIF4A3 interactions that are independent of its DEAD-box domain. Thus, analysis of long binding sites with iCLIP experiments can provide valuable insights into mechanisms of protein-RNA complexes.

## Conclusions

We find that the presence of non-coinciding cDNA-starts in iCLIP is not a signature of readthrough cDNAs, but instead reflects cDNA-end constraints. These can particularly affect the assignment of long binding sites of RBPs. To overcome these constraints, multiple technical aspects of iCLIP need to be optimised, including the conditions of RNase fragmentation, RNA ligation and cDNA purification. This produces cDNA libraries with a broad cDNA length distribution and low cDNA-end constraints, which are well suited for assigning the complete RNA binding sites of RBPs. These considerations apply to all protocols that amplify truncated cDNAs, including iCLIP, eCLIP and irCLIP, and they ensure that cDNA-starts comprehensively identify protein-RNA crosslink sites across the transcriptome. 

## Methods

### iCLIP experiments

iCLIP experiments are based on the previously described protocol [17] with minor modifications. In PTBP1-iCLIP1 (which was already used for a previous publication [12]), no antiRNase was used and the concentration of RNase I was 0.5 U/mL. In PTBP1-iCLIP2, 4SU was used for crosslinking as previously described [17] and the RNase conditions were optimised to ensure efficient RNase I-dependent fragmentation. In detail, HEK293T cells were grown on 10 cm^2^ dishes, incubated for 8 h with 100 μM 4SU and crosslinked with 2× 400 mJ/cm^2^ 365 nm UV light. Protein A Dynabeads were used for immunoprecipitations (IP). Eighty microlitres of beads were washed in iCLIP lysis buffer (50 mM Tris-HCl pH 7.4, 100 mM NaCl, 1% NP-40, 0.1% SDS, 0.5% sodium deoxycholate). For the preparation of the cell lysate, 2 million cells were lysed in 1 mL of iCLIP lysis buffer and the remaining cell pellet was dissolved in 50 μL urea lysis buffer (50 mM Tris-HCl, pH 7.4, 100 mM NaH_2_PO_4_, 7 M urea, 1 mM DTT). After the pellet had dissolved, the mixture was diluted with CLIP lysis buffer to 1000 μL, an additional centrifugation was performed and the two lysates were pooled before proceeding (2 mL total volume). As control for purity of protein–RNA complexes, we used a high-RNase condition for analysis by SDS-PAGE gel, but not for further preparation of cDNA library (Additional file 1: Figure S1A). For the full experiment, we incubated 2 mL of lysate with 4 U/mL of RNase I and 2 μL antiRNase (1/1000, AM2690, Thermo Fisher) at 37 °C for 3 min and centrifuged (Additional file 1: Figure S1B). We took care to prepare the initial dilution of RNase in water, since we found that RNase I gradually loses its activity when diluted in the lysis buffer. In total, 1.5 mL of the supernatant was then added to the beads, incubated at 4 °C for 4 h and cDNA library was prepared based on the standard protocol. In PTBP1-iCLIP3, the dephosphorylation step was omitted from step 2 (Fig. 1a) and the rest was same as the standard protocol.

eIF4A3-iCLIP2 was performed from HEK293 cells crosslinked with 0.15 mJ/cm^2^ 254 nm UV light. To prepare the cell lysate, the cells were lysed with 1 mL iCLIP lysis buffer (50 mM Tris-HCl pH 7.4, 100 mM NaCl, 1% NP-40, 0.1% SDS, 0.5% sodium deoxycholate, final concentration 2 mg/mL) and sonicated (Bioruptor, 5 × 5 s on/off). Two replicates were produced, one with 1 U/mL and the other with 2 U/mL of RNase I in 1 mL of lysate. The SDS-PAGE analysis showed the size of the resulting protein-RNA complexes to be similar (Additional file 1: Figure S1C) and therefore we grouped these two replicates for all analyses of eIF4A3-iCLIP2. After RNase treatment, the samples were centrifuged. For each IP, 100 μL of Protein G Dynabeads were washed in iCLIP lysis buffer and incubated with the anti-eIF4A3 antibody produced in the Le Hir laboratory [11]. The samples were rotated at 4 °C for 2 h. The beads were then washed with high-salt washing buffer (50 mM Tris-HCl pH 7.4, 1 M NaCl, 1% NP-40, 0.1% SDS, 0.5% sodium deoxycholate). After the first round of washes, the samples proceed through 3′ adapter addition, an additional phosphorylation (0.2 μL PNK, 0.4 μL cold ATP [1 mM], 0.4 μL 10× PNK buffer, 3 μL water). After SDS-PAGE separation, the guidelines recommend isolating radioactive RBP-RNA complexes that migrate 20–100 kDa higher than the RBP alone, which leads to isolation of RNA molecules of 50–300 nt. Since we expect that most cDNAs truncate at crosslink sites within these RNA molecules, we prepared the iCLIP library with a heterogeneous population of cDNAs that were 30–140 nt long (Additional file 1: Figure S1D). We then produced sequence reads of 150 nt using the Illumina MiSeq platform for PTBP1-iCLIP2 and 120 nt using the Illumina HiSeq platform for eIF4A3-iCLIP2, thereby obtaining sequences for cDNAs up to a length of 139 or 109 nt, respectively (after removal of adapters).

eIF4A3-iCLIP3 was performed from HeLa cells based on the previously described protocol [17] with minor modifications. Briefly, HeLa cells were grown on 10 cm^2^ dishes and crosslinked with 0.15 mJ/cm^2^ 254 nm UV light. Protein A Dynabeads were used for IPs. For each IP, 40 μL of beads were washed in iCLIP lysis buffer and incubated with 5 μL of anti-eIF4A3 antibody produced in the Le Hir laboratory [11]. For the preparation of the cell lysate, the cells were scraped from a 10 cm^2^ dish and lysed in 1 mL of iCLIP lysis buffer, incubated with of 1 U/mL of RNase I at 37 °C for 3 min and centrifuged. The supernatant was then added to the beads and incubated at 4 °C for 2 h. Afterwards, the beads were washed with IP buffer (10 mM Tris, 150 mM NaCl, 2.5 mM MgCl_2_, 1% NP-40), RIPA-S buffer (50 mM Tris, 1 M NaCl, 5 mM EDTA, 2 M urea, 0.5% NP-40, 0.1% SDS, 1% sodium deoxycolate) and PNK buffer before proceeding to the iCLIP protocol for cDNA library preparation and Illumina HiSeq sequencing produced 50 nt sequence reads (Additional file 1: Figure S1E, F).

### Computational analyses

All the source codes used for the analyses in this paper are released under an open source license compliant with OSI (http://opensource.org/licenses) and are available at the GitHub (https://github.com/jernejule/non-coinciding_cDNA_starts) and Zenodo repository (https://zenodo.org/badge/latestdoi/57377213).

### Trimming of the adapter sequences

Before mapping the cDNAs, we removed unique molecular identifiers (UMIs) and trimmed the 3′ Solexa adapter sequence. Adapter sequences were trimmed with the FASTX-Toolkit 0.0.13 adapter removal software, using the following parameters: -Q 33 -a AGATCGGAAG -c -n -l 26.

### Mapping of iCLIP sequence data

To map iCLIP sequence data for PTBP1 and all RBPs other than eIF4A3, we used the UCSC hg19/GRCh37 genome assembly and the Bowtie2 version 2.1 alignment software with default settings. More than 81% (1,642,850 of 2,007,824) and 85% (8,585,142 out of 9,634,025) of all cDNAs from the published and newly generated iCLIP data, respectively, mapped uniquely to a single genomic position. To map the eIF4A3 iCLIP data, we compiled a set of the longest mRNA sequence available for each multi-exon gene from BioMart Ensembl Genes 79. We mapped to these mRNAs with the Bowtie2 version 2.1 alignment software, allowing two mismatches. More than 50% (11,935,475 of 23,040,243) of cDNAs from all eIF4A3 iCLIP datasets mapped to a unique mRNA position.

The first 9 nt of the sequenced iCLIP read correspond to the barcode, which contains the experimental identifier that allows to separate experimental replicates, and the UMIs, which allow to avoid artefacts caused by variable PCR amplification of different cDNAs (Fig. 1a, step 8, orange) [3]. We used these UMIs to quantify the number of unique cDNAs that mapped to each position in the genome (for PTBP1) or transcriptome (for eIF4A3) by collapsing cDNAs with the same UMI that mapped to the same starting position to a single cDNA.

### Definition of crosslink-associated (CL) motifs

We reasoned that sequence motifs enriched directly at the starts of the mock eCLIP cDNAs might uncover preferences of UV crosslinking, since they are thought to represent a mixture of crosslink sites for many different RBPs and thus they should not reflect sequence specificity of any specific RBP [6]. We therefore examined occurrence of tetramers that overlapped with the nucleotide preceding the cDNA-starts (position –1 nt) in comparison with the ones overlapping with the 10th nucleotide preceding the cDNA-starts (position –10 nt) in PTBP1 mock input eCLIP. We excluded the TTTT tetramer from further analyses, since it is often part of longer tracts of Ts, and therefore its inclusion decreases the resolution of analysis. Thus, the tetramers that are enriched over 1.5-fold at position –1 compared to –10 include TTTG, TTTC, TTGG, TTTA, ATTG, ATTT, TCGT, TTGA, TTCT and CTTT, and these were considered for all analyses of ‘CL-motifs’.

### Definition of crosslink clusters

The crosslink clusters were identified by False Discovery Rate peak finding algorithm from iCount (https://github.com/tomazc/iCount), by assessing the enrichment of cDNA-starts at specific sites compared to shuffled data as described previously [30], with the following additional details. At each cDNA-start, the counts of all cDNAs containing their cDNA-start at a maximum spacing of 15 nt were summed up (or at 3 nt spacing for Fig. 5e). Then crosslink clusters were defined by using the cDNA-starts with counts that passed the false discovery rate < 0.05 significance threshold (determined by comparing the count distribution to shuffled data). Neighbouring clusters that were less than 21 nt apart (or 3 nt apart for Fig. 5e) were then merged into single clusters.

### Definition of cDNA-start peak and cDNA-end peak

The peak position of cDNA-starts was identified by comparing the counts at each cDNA-start and choosing the position with the maximum count within each defined region from the top 1000 exon-exon junctions that contain the highest number of cDNAs. Peak positions with a low number of cDNAs (less then median number of all top cDNA-start peaks) were ignored. If more than one position of cDNA-starts had equal count, then the position with maximum count that was located closest to the start of the defined region was chosen. The same approach was used to define cDNA-end peaks.

### Definition of PTBP1-binding motifs

To identify the motifs bound by PTBP1, we searched for pentamers enriched in the region [–10..10] around the cDNA-start peaks identified in each crosslink cluster defined by PTBP1-iCLIP2. Sixty-nine pentamers had enrichment z-score > 299 and were used as PTBP1-binding pentamers for further analyses. Their sequences are: TCTTT, CTTTC, TCTTC, CTTCT, TCTCT, CTCTC, TTTCT, TTCTC, TTCTT, TTTTC, TCCTT, CTCTT, ATTTC, TTCCT, CTTCC, TTTCC, CCTTT, CTTTT, CCTTC, TCTGT, TTCTG, TCCTC, CTTCA, ATCTT, TGTCT, TCTGC, CTCCT, CCTCT, GTCTT, TCTAT, TCTCC, ATTCC, TTCTA, CTTTG, TATCT, ACTTC, TTATC, CTTAT, CTATT, TTCAT, TTCCA, TCTTG, TTGTC, TTGCT, CTCTA, CTCTG, TATTT, TCCCT, TCATT, TTCCC, CATTT, ATTCT, TTTAC, GTTCT, CTATC, TCATC, CTTTA, TGTTC, TATTC, CATCT, TACTT, CTGTT, CTTGC, ACCTT, TTTCA, TTTGT, TGTTT, CTTGT, ACTTT. All of these pentamers are enriched in pyrimidines, in agreement with the known preference of PTBP1 for UC-rich binding motifs [31].

### Visualisation of cDNA distributions with the density graphs (used in Figs. **4**a–d, **5**a–d, **6**a–f, **7**a and b, Additional file **1**: Figure **3**A–E, Additional file **1**: Figure **4**I–K, Additional file **1**: Figure **5**A and B)

All normalisations were performed in R (version 3.1.0) together with the ‘ggplot2’ and the ‘smoother’ package for the final graphical output. For the analysis of eIF4A3 iCLIP, each density graph (RNA map) shows a distribution of cDNA-starts and cDNA-ends relative to positions of exon-exon junctions or end peaks in mRNAs. To avoid any border effects, we examined only exon-exon junctions within coding regions, excluding the first or the last junction. The number of cDNAs starting or ending at each position on the graph was normalised by the number of all cDNAs mapped to representative mRNAs, the mRNA length and the number of examined exon-exon junction positions, as described below:

$$ \mathrm{RNAmap}\left[\mathrm{n}\right] = \left(\left(\mathrm{cDNAs}\left[\mathrm{n}\right]/\mathrm{sum}\left(\mathrm{cDNAs}\right)\right)\ *\ \mathrm{length}\left(\mathrm{mRNAs}\right)/\mathrm{count}\left(\mathrm{exon}\_\mathrm{exon}\_\mathrm{junctions}\right)\right. $$

where [n] stands for a specific position on the density graph.

To draw the graph, we then used the Gaussian method with a 5-nt smoothing window.

For the analysis of PTBP1 iCLIP and CLIP, each density graph (RNA map) shows a distribution of cDNA-starts and cDNA-ends relative to positions of its binding sites, which were defined using the position of Y-tracts. We obtained genomic positions of all TC-rich and T-rich low complexity sequences that are present in introns in protein-coding genes in the human genome by using the UCSC table browser.

To avoid the effects of variable abundance of intronic RNAs (and occasional presence of highly abundant non-coding transcripts, such as snoRNAs), we then normalised counts at each binding site by the density of cDNAs in the same region. For this purpose, we examined the region of the binding site, as well as 120 nt 5′ and 3′ of the binding site, to find the nucleotide with the largest count of cDNA-starts or ends (depending on whether starts or ends were plotted on the graph), which is referred to as ‘MaxCount’. We thus obtained ‘MaxCount-normalised cDNA counts’ at each position (which were between 0 and 1). For drawing RNA maps, we wished to examine the enrichment of cDNA counts within binding sites compared to nearby regions outside of binding sites. We therefore calculated the average ‘MaxCount-normalised cDNA counts’ at each position across the evaluated binding sites and divided this count at each position by the average ‘MaxCount-normalised cDNA counts’ in the region 50–100 nt downstream of the binding site, as described in the formula below:

$$ \mathrm{RNAmap}\left[\mathrm{n}\right] = \mathrm{average}\ \mathrm{normalised}\ \mathrm{cDNAs}\left[\mathrm{n}\right]/\ \mathrm{average}\ \mathrm{normalised}\ \mathrm{cDNAs} \times \left[50-100\ \mathrm{nt}\ \mathrm{downstream}\ \mathrm{of}\ \mathrm{the}\ \mathrm{binding}\ \mathrm{site}\right] $$

where [n] stands for a specific position on the density graph.

To draw the graph, we then used the Gaussian method with a 10-nt smoothing window. The empirical cumulative distribution (Fig. 4a, c) were generated in R with stat_ecdf function from ggplot2 package by using frequency of raw cDNA-start counts for each length category in region 25 nt upstream and downstream from cDNA start peak.

### Assignment of the cDNA-end peak in eIF4A3 iCLIP

For cDNA-end peak assignment in eIFA3 iCLIP data, we used exons longer than 100 nt that were in the top 50% of the distribution of exons based on cDNA coverage. This ensured that sufficient cDNAs were available for assignment of the putative binding sites. We then summarised all cDNA-end positions in the region –20 to +25 around exon-exon junctions and selected the position with the maximum cDNA count as the ‘cDNA-end peak’.

### Analysis of pairing probability

Computational prediction of the secondary structure around the cDNA-end peaks was performed using the RNAfold program with the default parameters [32].

### Analysis of cDNA transitions

Density of C-to-T transitions across cDNAs was performed by using the samtools software with the following parameters: samtools calmd –u –u genomic.fasta input_BAM > BAM_with_transitions. This pipeline replaces BAM format mapped cDNA sequences with transitions relative to genomic reference. In the next step of the following pipeline we used a custom python script (available on github repository) that returns a density array of C-to-T transitions for cDNAs that are shorter than 40 nt. For the final visualisation of density graphs, we used the same approach as for all other density figures without additional normalisations.

## Additional file

Additional file 1: Figure S1. Quality control of the PTBP1 and eIF4A3 iCLIP. **Figure S2.** CL-motifs are enriched at cDNA deletions and cDNA-starts in U2AF2-iCLIP. **Figure S3.** Analysis of cDNA-starts and cDNA-ends at the start of Y-tracts. **Figure S4.** Constrained cDNA-ends in eIF4A3 iCLIP. **Figure S5.** The impact of cDNA-end constraints on cDNA-starts in eIF4A3 iCLIP. **Figure S6.** Distribution of cDNA sizes in the studied experiments. (PDF 5.42 mb)
