# Erratum to: Insights into the design and interpretation of iCLIP experiments

**DOI:** 10.1186/s13059-017-1294-z

**Published:** 2017-08-15

**Authors:** Nejc Haberman, Ina Huppertz, Jan Attig, Julian König, Zhen Wang, Christian Hauer, Matthias W. Hentze, Andreas E. Kulozik, Hervé Le Hir, Tomaž Curk, Christopher R. Sibley, Kathi Zarnack, Jernej Ule

**Affiliations:** 1 Department of Molecular Neuroscience, UCL Institute of Neurology, Queen Square, London, WC1N 3BG UK; 2 The Crick Institute, 1 Midland Road, London, NW1 1AT UK; 3 MRC Laboratory of Molecular Biology, Francis Crick Avenue, Cambridge, CB2 0QH UK; 4 European Molecular Biology Laboratory (EMBL), Meyerhofstrasse 1, 69117 Heidelberg, Germany; 5 Institute of Molecular Biology (IMB), Ackermannweg 4, 55128 Mainz, Germany; 6 Institut de Biologie de l’ENS (IBENS), Paris, France; 7 CNRS UMR 8197, Paris, Cedex 05 75230 France; 8 Molecular Medicine Partnership Unit (MMPU), Im Neuenheimer Feld 350, 69120 Heidelberg, Germany; 9 Department of Pediatric Oncology, Hematology and Immunology, University of Heidelberg, Im Neuenheimer Feld 430, 69120 Heidelberg, Germany; 10 Faculty of Computer and Information Science, University of Ljubljana, Tržaška cesta 25, 1000 Ljubljana, Slovenia; 11 Division of Brain Sciences, Department of Medicine, Imperial College London, London, UK; 12 Buchmann Institute for Molecular Life Sciences (BMLS), Goethe University Frankfurt, Max-von-Laue-Str. 15, 60438 Frankfurt, Germany

## Erratum

Upon publication of the original article [1], it was noted that data submitted by the authors was accidentally omitted during typesetting. The following section of the submitted manuscript was wrongfully omitted from the Methods section in the published article [1]:

## Availability of data and materials

All iCLIP data that were newly generated for this manuscript are made available at http://www.ebi.ac.uk/arrayexpress/ via accession numbers E-MTAB-5027 (PTBP1-iCLIP2), E-MTAB-5026 (PTBP1-iCLIP3), E-MTAB-3618 (eIF4A3-iCLIP2) and E-MTAB-4000 (eIF4A3-iCLIP3).

This has now been acknowledged and corrected in this erratum.

The publisher apologises for these errors.
