# Supplementary material for: Insights into the design and interpretation of iCLIP experiments
Source: Genome Biol. 2017 Jan 16;18:7. doi: 10.1186/s13059-016-1130-x (PMC5240381; doi:10.1186/s13059-016-1130-x)

Insights into the design and interpretation of iCLIP experiments

Haberman N[1,2], Huppertz I[1,3,4], Attig J[1,2], König J[1,5], Wang Z[6,7], Hauer C[4,8,9], Hentze MW[4,8], Kulozik AE[8,9], Le Hir H[6,7], Curk T[10], Sibley CR[1,11], Zarnack K[12], Ule J[1,2]

[1]Department of Molecular Neuroscience, UCL Institute of Neurology, Queen Square, London, WC1N 3BG, UK

[2]The Crick Institute, 1 Midland Road, London NW1 1AT, UK

[3]MRC Laboratory of Molecular Biology, Francis Crick Avenue, Cambridge, CB2 0QH, UK

[4]European Molecular Biology Laboratory (EMBL), Meyerhofstrasse 1, 69117 Heidelberg, Germany

[5]Institute of Molecular Biology (IMB), Ackermannweg 4, 55128 Mainz, Germany

[6]Institut de Biologie de l'ENS (IBENS), 46 rue d'Ulm, Paris F-75005, France

[7]CNRS UMR 8197, Paris Cedex 05, 75230, France

[8]Molecular Medicine Partnership Unit (MMPU), Im Neuenheimer Feld 350, 69120 Heidelberg, Germany

[9]Department of Pediatric Oncology, Hematology and Immunology, University of Heidelberg, Im Neuenheimer Feld 430, 69120 Heidelberg, Germany

[10]Faculty of Computer and Information Science, University of Ljubljana, Tržaška cesta 25, SI-1000, Ljubljana, Slovenia

[11]Division of Brain Sciences, Department of Medicine, Imperial College London, London, UK.

[12]Buchmann Institute for Molecular Life Sciences (BMLS), Goethe University Frankfurt, Max-von-Laue-Str. 15, 60438 Frankfurt am Main, Germany

Author Information:

Nejc Haberman and Ina Huppertz contributed equally to this work.

Corresponding authors:

Jernej Ule: j.ule@ucl.ac.uk

Kathi Zarnack: kathi.zarnack@bmls.de

Keywords: protein-RNA interactions, iCLIP, eCLIP, irCLIP, binding site assignment, high-throughput sequencing, PTBP1, eIF4A3, exon-junction complex

Additional file 1: Figures S1 to S6

Figure S1 | Quality control of the PTBP1 and eIF4A3 iCLIP.

a) SDS-PAGE analysis of PTBP1-iCLIP2 performed with high RNase conditions. X-ray film shows exposure to the nitrocellulose membrane harbouring PTBP1-RNA complexes that were separated on SDS-PAGE. Size range of the isolated complexes is indicated on the left. MW, molecular weight.

b) Same as a), but performed with low RNase conditions.

c) Same as a), but showing eIF4A3-RNA complexes isolated in the eIF4A3-iCLIP2 experiment.

d) Native acrylamide gel showing eIF4A3-iCLIP2 PCR products that were amplified from medium (M) and high (H) cDNA size ranges. During cDNA library preparation, two size ranges of cDNAs were isolated from the denaturing acrylamide gel as in the recommended protocol [5]. After amplification, 170-270 nt PCR products were obtained, including 128 nt of Illumina primer and iCLIP barcodes, corresponding to an approximate size range of mappable sequenced cDNAs of 40-140 nt.

d) Same as a), but this time showing eIF4A3-RNA complexes isolated in the eIF4A3-iCLIP3 experiment.

e) Same as d), but this time showing eIF4A3-iCLIP3 PCR products.

Figure S2 | CL-motifs are enriched at cDNA deletions and cDNA-starts in U2AF2-iCLIP.

a) Analysis of all U2AF2 experiments examined in this study shows the proportion of cDNAs from each experiment that overlap with a CL-motif at each position relative to the cDNA-start.

b) Analysis of all eIF4A3 experiments examined in this study shows the proportion of cDNAs from each experiment that overlap with a CL-motif at each position relative to the cDNA-start.

c) Proportion of PTBP1-iCLIP1 cDNAs that overlap with a CL-motif at each position relative to the cDNA-start. Only cDNAs shorter than 40 nt are examined, and are divided into those lacking deletions, or containing a deletion within the first 7 nt, or anywhere in the remaining portion of the cDNA.

d) Same as c), but for U2AF2-iCLIP.

Figure S3 | Analysis of cDNA-starts and cDNA-ends at the start of Y-tracts.

a) The cDNA-starts of PTBP1-iCLIP1 and CLIP experiments are plotted around the starts of >35 nt Y-tracts that are annotated as T-rich or TC-rich low-complexity sequence in the human genome (hg19). cDNAs of PTBP1-iCLIP1 are divided into four length categories: 17-29 nt, 30-34 nt, 35-39 nt, and >39 nt.

b) Same as a), but using U2AF2-iCLIP and CLIP cDNAs.

c) Same as a), but showing the positions of cDNA-ends**.**

d) Same as b), but showing the positions of cDNA-ends**.**

e) Same as a), but for the cDNA-centres around the ends of Y-tracks.

f) Ratio of cDNA-starts and cDNA-centres that are inside of Y-tracks compared to the downstream region (schematic description at the bottom). Statistical test for cDNA-starts and cDNA-centres enrichment in Y-tracts region was done by Fisher's Exact Test with p-value < 2.2e-16.

Figure S4 | Constrained cDNA-ends in eIF4A3 iCLIP.

a) Heatmap showing the position of cDNA-ends around top 1,000 exon-exon junctions contain the highest number of cDNAs in eIF4A3-iCLIP1. Junctions are grouped in each row based on their shared position of cDNA-end peaks (marked by blue rectangle). Each row shows the average of cDNA counts across the grouped junctions. The values are normalised against the maximum value across all rows.

b) Same as a), but for eIF4A3-iCLIP2.

c) Same as a), but showing summarised pairing probability around exon-exon junctions in eIF4A3-iCLIP1. The position of the cDNA-end peak is marked by the white rectangle.

d) Same as c), but for eIF4A3-iCLIP2.

e) Genomic nucleotide composition around cDNA-end peaks in eIF4A3-iCLIP1.

f) Same as e), but for eIF4A3-iCLIP2.

g) Same as e), but for eIF4A3-iCLIP3.

h) Same as e), but for eIF4A3-CLIP.

i-k) Distribution of cDNA-starts (solid lines) and ends (dotted lines) in eIF4A3-iCLIP2 relative to exon-exon junctions. Junctions were divided into three different classes according to the position cDNA-end peaks at: -7 to 2 nt **(i)**, 3 to 12 nt **(j)**, or 13 to 25 nt **(k)** relative to exon-exon junctions. cDNA length categories and labelling as shown on top.

Figure S5 | The impact of cDNA-end constraints on cDNA-starts in eIF4A3 iCLIP.

a) The distribution of cDNA-starts (solid lines) and ends (dotted lines) relative to the cDNA-end peaks that were identified at top 1,000 exon-exon junctions contain the highest number of cDNAs in eIF4A3-iCLIP1. cDNAs are divided into three length categories: 17-29 nt, 30-34 nt, and 35-39 nt.

b) Same as a), but for eIF4A3-iCLIP3.

c) Same as a), but for the junction that ranks 8th by the number of cDNAs in eIF4A3-iCLIP1.

d) Same as b), but for the junction that ranks 1st by the number of cDNAs in eIF4A3-iCLIP3.

e) Same as a), but for the junction that ranks 14th by the number of cDNAs in eIF4A3-iCLIP1.

f) Same as b), but for the junction that ranks 4th by the number of cDNAs in eIF4A3-iCLIP3.

g) Same as a), but for the junction that ranks 10th by the number of cDNAs in eIF4A3-iCLIP1.

h) Same as b), but for the junction that ranks 5th by the number of cDNAs in eIF4A3-iCLIP3.

Figure S6 | Distribution of cDNA sizes in the studied experiments.

a) Distribution of cDNA sizes in eIF4A3 CLIP and iCLIP experiments of cDNAs that are shorter than 39 nt. The number above the lines reports the % of cDNAs shorter than 39 nt. For longer cDNAs, it is not possible to draw the distribution as their precise lengths are unknown due to the limited length of sequencing. Thus, both the distribution and the % needs to be taken into account to estimate if there is a narrow distribution of cDNA sizes. For example, the distribution shows preferred lengths for both eIF4A3-iCLIP1 and eIF4A3-iCLIP3, but in case of eIF4A3-iCLIP3 only 36% of cDNAs are shorter than 39 nt, while in eIF4A3-iCLIP1 approximately 50% of cDNAs are in the length range of 27-37 nt. Thus, only eIF4A3-iCLIP1 has a strong potential for the cDNA distribution to affect binding site assignment.

b) Same as a), but for PTBP1 CLIP experiments, showing the % of cDNAs shorter than 34 nt due to the shorter sequencing length.

c) Same as b), but for U2AF2-iCLIP which shows a trend for shorter cDNA size distribution, with 54% of cDNAs <39 nt. However, this is not a major problem due to the lesser cDNA-end constraints in this experiment (see the broad distribution of cDNA-ends around intron-exon junctions in Figure 6a).

Figure S1

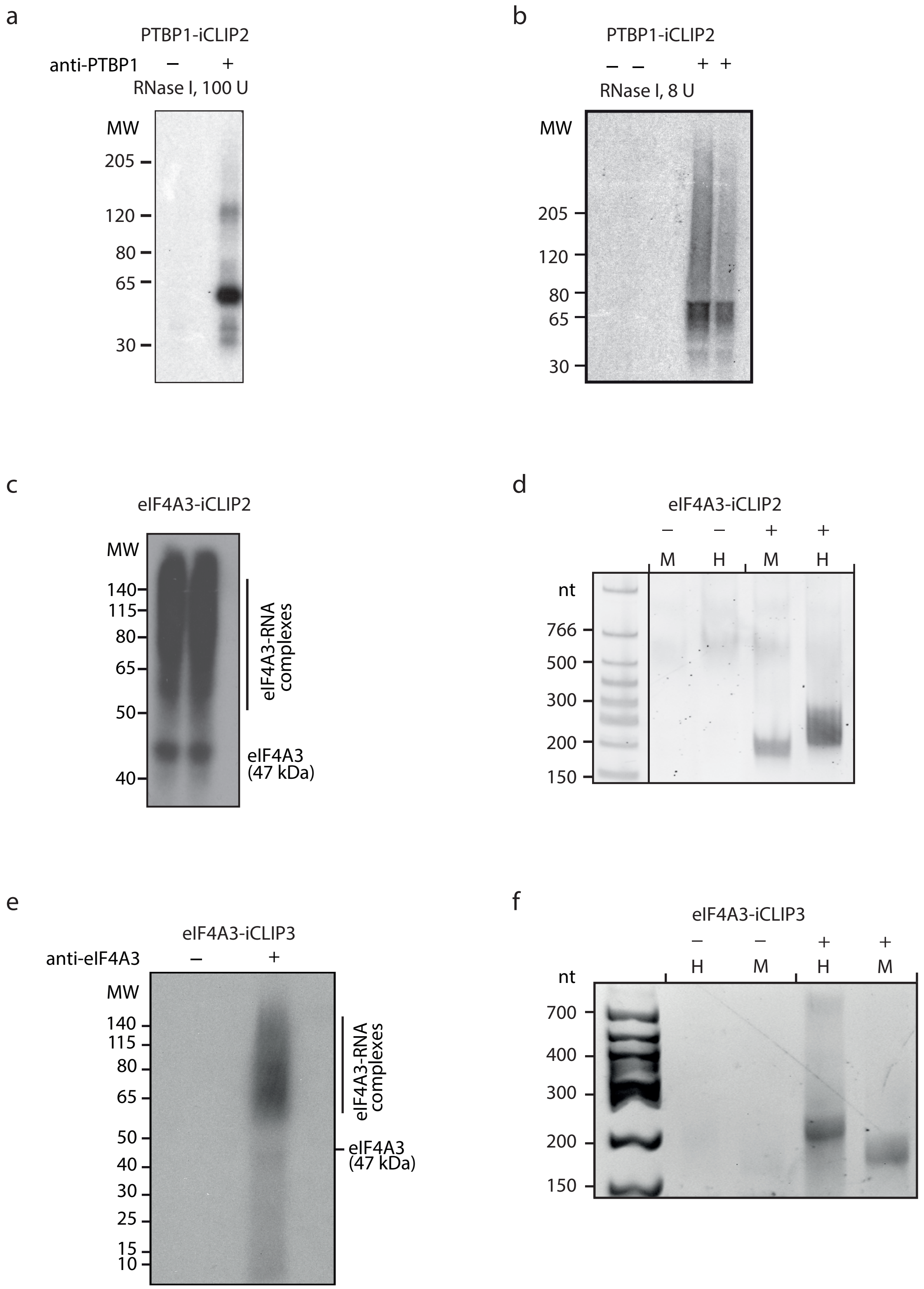

a
PTBP1-iCLIP2
anti-PTBP1 – +
RNase I, 100 U
MW
205
120
80
65
30
b
PTBP1-iCLIP2
– – + +
RNase I, 8 U
MW
205
120
80
65
30
c
eIF4A3-iCLIP2
MW
140
115
80
65
50
40
eIF4A3-RNA complexes
eIF4A3 (47 kDa)
d
eIF4A3-iCLIP2
– – + +
M H M H
nt
766
500
300
200
150
e
eIF4A3-iCLIP3
anti-eIF4A3 – +
MW
140
115
80
65
50
40
30
25
15
10
eIF4A3-RNA complexes
eIF4A3 (47 kDa)
f
eIF4A3-iCLIP3
– – + +
H M H M
nt
700
400
300
200
150

Figure S2

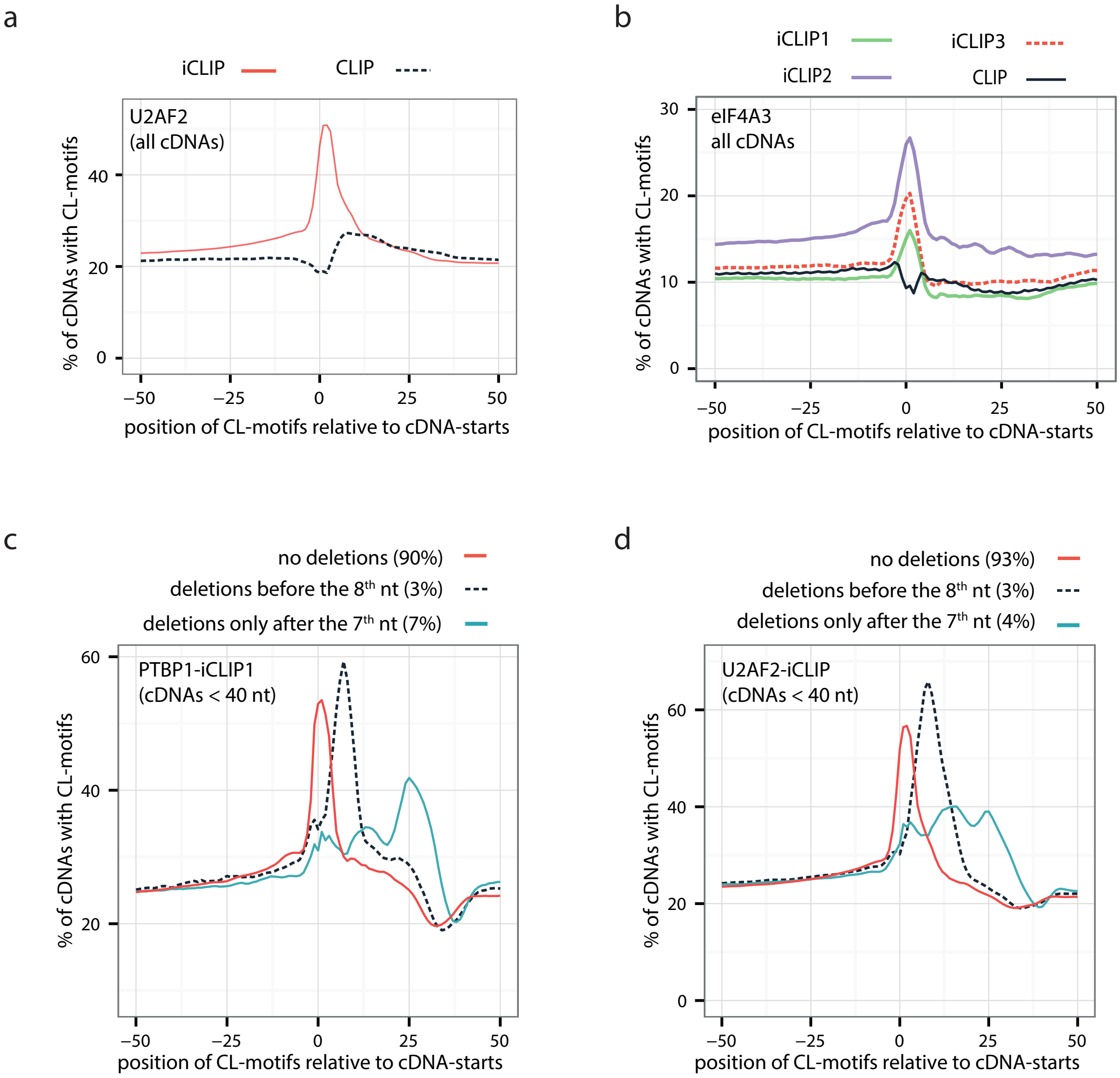

a
iCLIP
CLIP
U2AF2
(all cDNAs)
% of cDNAs with CL-motifs
0
20
40
−50
−25
0
25
50
position of CL-motifs relative to cDNA-starts
b
iCLIP1
iCLIP2
iCLIP3
CLIP
eIF4A3
all cDNAs
% of cDNAs with CL-motifs
0
10
20
30
−50
−25
0
25
50
position of CL-motifs relative to cDNA-starts
c
no deletions (90%)
deletions before the 8th nt (3%)
deletions only after the 7th nt (7%)
PTBP1-iCLIP1
(cDNAs < 40 nt)
% of cDNAs with CL-motifs
20
40
60
−50
−25
0
25
50
position of CL-motifs relative to cDNA-starts
d
no deletions (93%)
deletions before the 8th nt (3%)
deletions only after the 7th nt (4%)
U2AF2-iCLIP
(cDNAs < 40 nt)
% of cDNAs with CL-motifs
0
20
40
60
−50
−25
0
25
50
position of CL-motifs relative to cDNA-starts

Figure S3

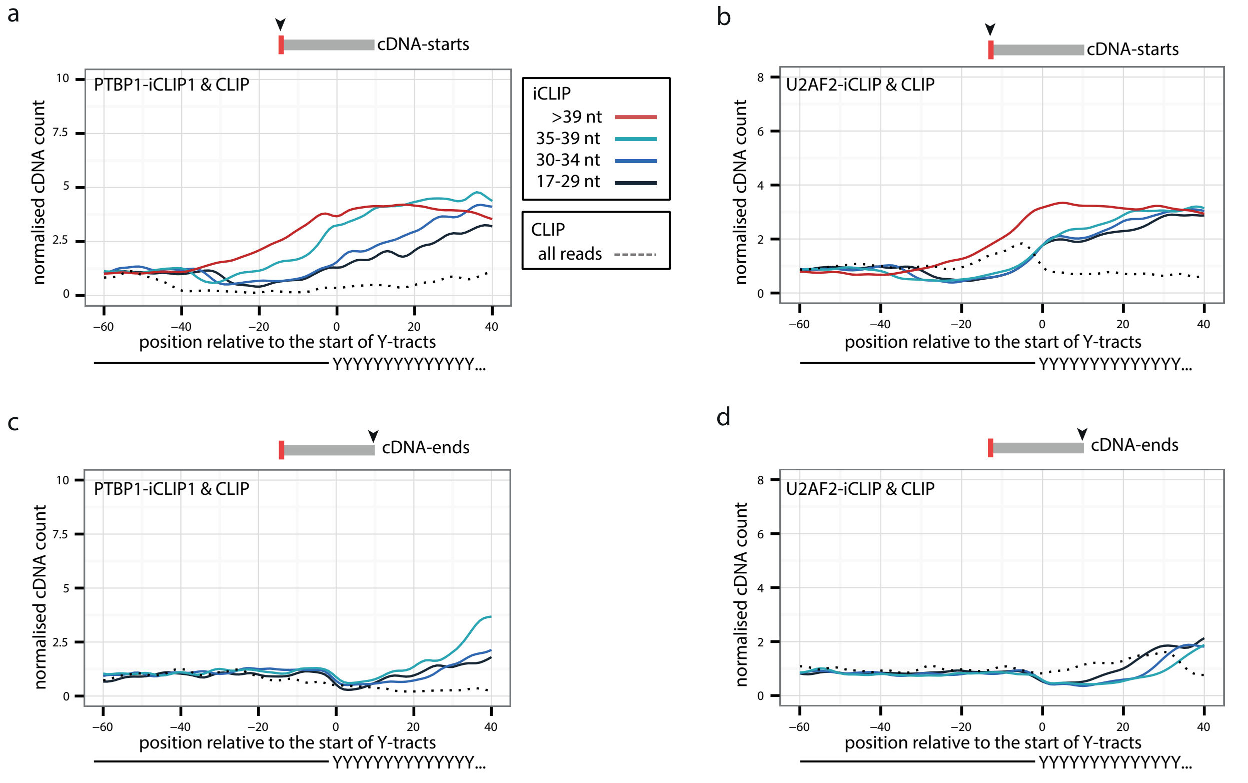

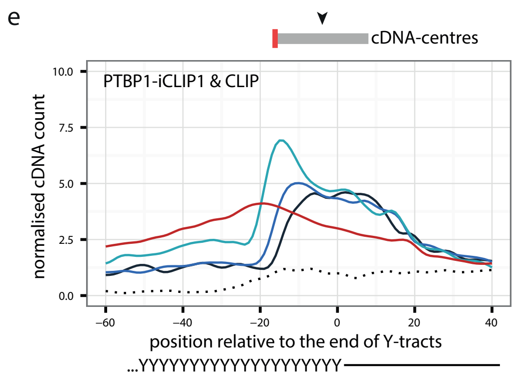

f

PTBP1-iCLIP1	cDNA-starts	cDNA-centres
inside Y-tracts	142,336	133,655
downstream Y-tracts	28,588	44,579
ratio	4.978872	2.998161

Fisher's Exact Test: p-value < 2.2e-16

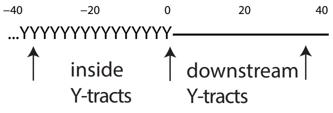

Figure S4

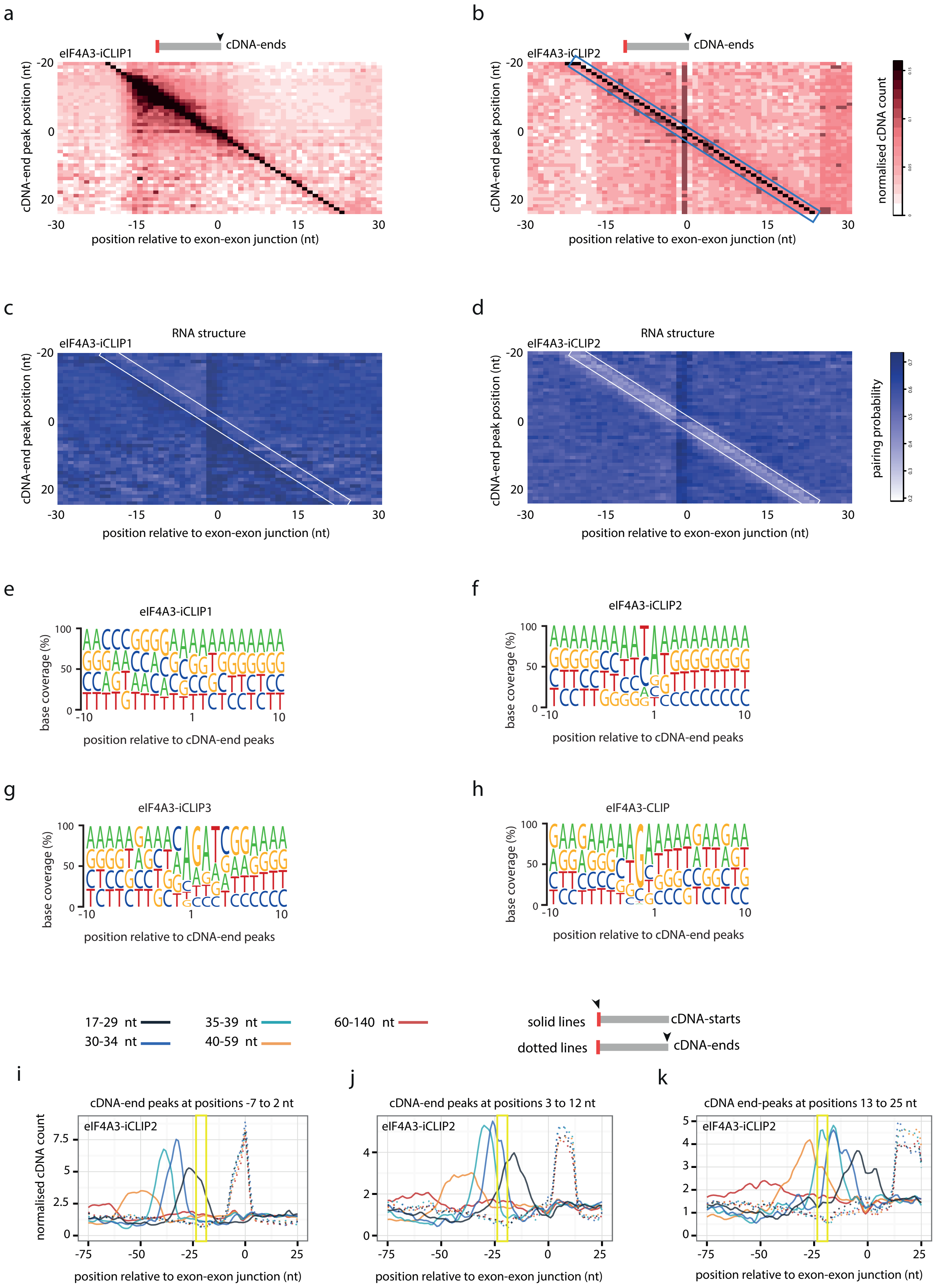
a
eIF4A3-iCLIP1
cDNA-ends
cDNA-end peak position (nt)
position relative to exon-exon junction (nt)
b
eIF4A3-iCLIP2
cDNA-ends
normalised cDNA count
c
RNA structure
eIF4A3-iCLIP1
d
RNA structure
eIF4A3-iCLIP2
pairing probability
e
eIF4A3-iCLIP1
base coverage (%)
position relative to cDNA-end peaks
f
eIF4A3-iCLIP2
g
eIF4A3-iCLIP3
h
eIF4A3-CLIP
17-29 nt
30-34 nt
35-39 nt
40-59 nt
60-140 nt
solid lines
cDNA-starts
dotted lines
cDNA-ends
i
cDNA-end peaks at positions -7 to 2 nt
j
cDNA-end peaks at positions 3 to 12 nt
k
cDNA end-peaks at positions 13 to 25 nt
normalised cDNA count

Figure S5

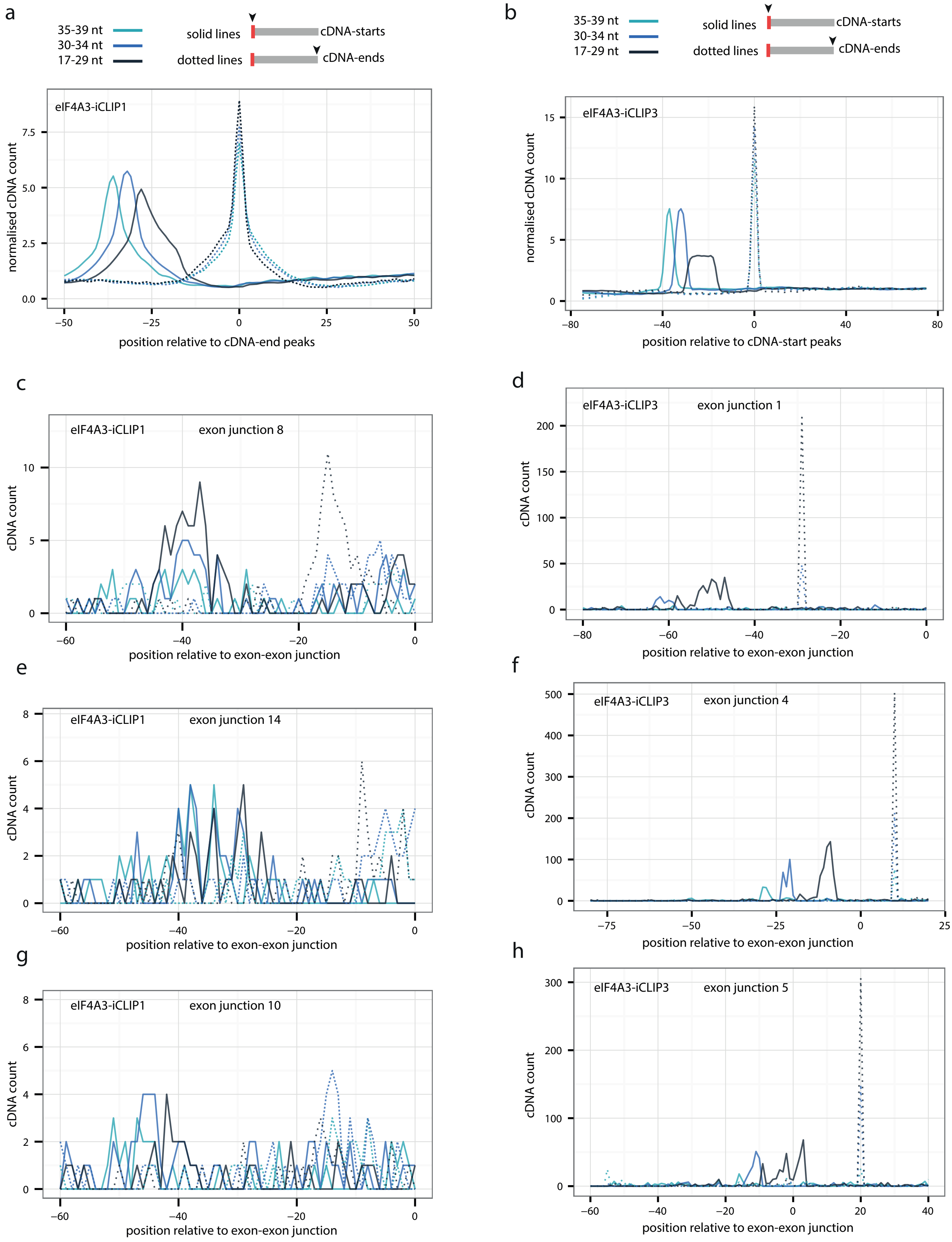
a
35-39 nt
30-34 nt
17-29 nt
solid lines
cDNA-starts
dotted lines
cDNA-ends
eIF4A3-iCLIP1
normalised cDNA count
position relative to cDNA-end peaks
b
eIF4A3-iCLIP3
normalised cDNA count
position relative to cDNA-start peaks
c
eIF4A3-iCLIP1
exon junction 8
cDNA count
position relative to exon-exon junction
d
eIF4A3-iCLIP3
exon junction 1
cDNA count
position relative to exon-exon junction
e
eIF4A3-iCLIP1
exon junction 14
cDNA count
position relative to exon-exon junction
f
eIF4A3-iCLIP3
exon junction 4
cDNA count
position relative to exon-exon junction
g
eIF4A3-iCLIP1
exon junction 10
cDNA count
position relative to exon-exon junction
h
eIF4A3-iCLIP3
exon junction 5
cDNA count
position relative to exon-exon junction

Figure S6

a

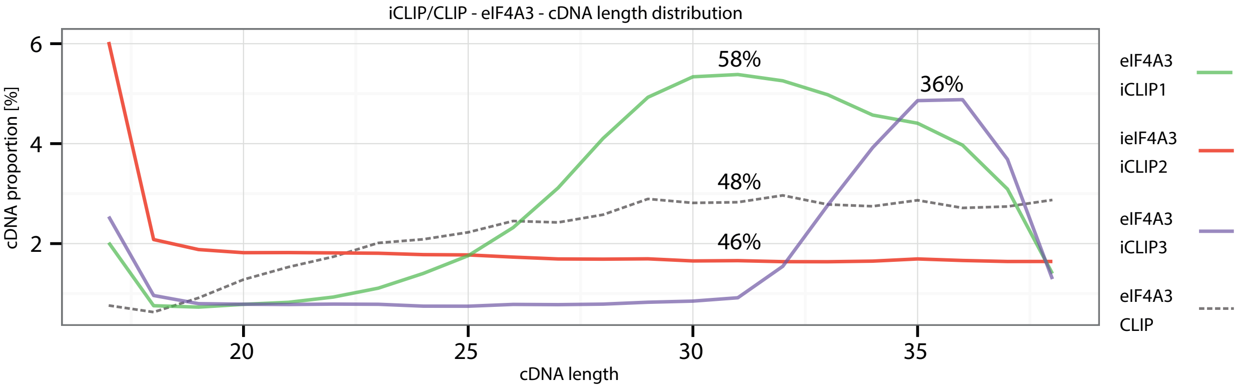

b

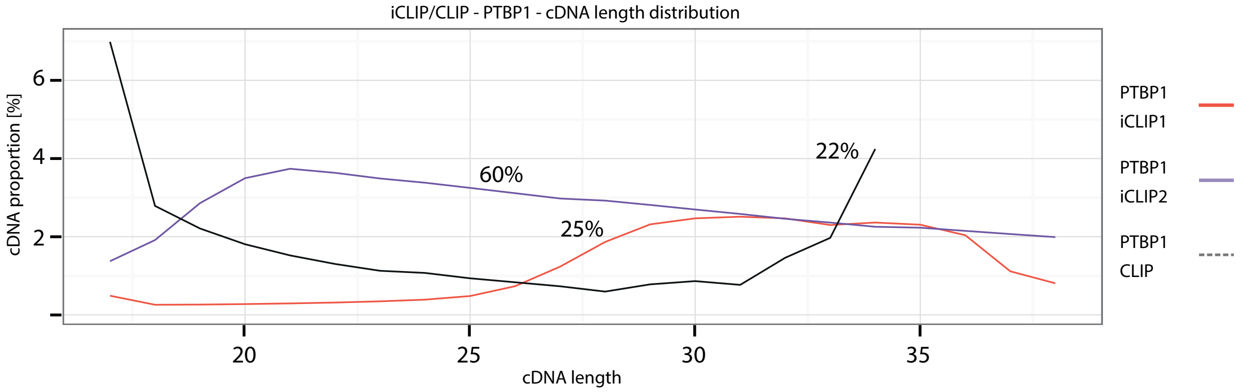

c

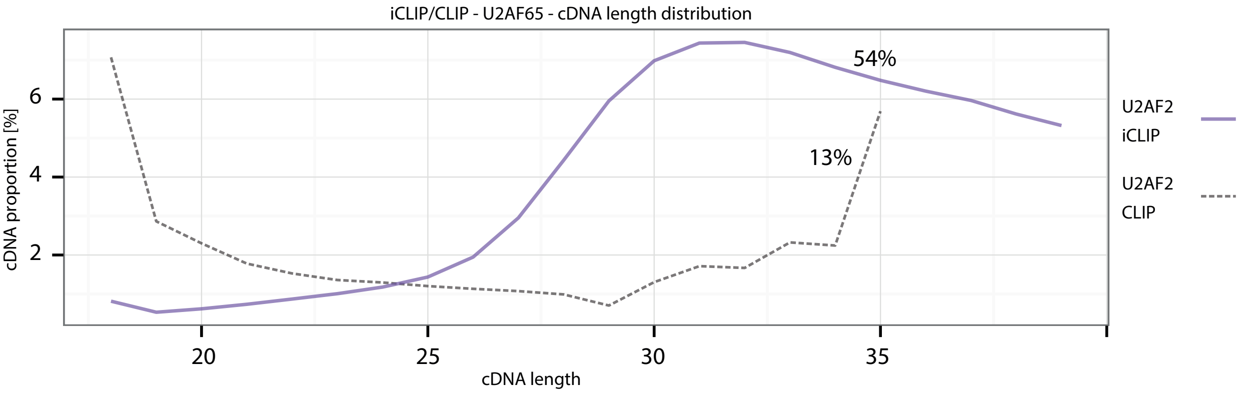

Supplement: Additional file 1: Figure S1. — Quality control of the PTBP1 and eIF4A3 iCLIP. Figure S2. CL-motifs are enriched at cDNA deletions and cDNA-starts in U2AF2-iCLIP. Figure S3. Analysis of cDNA-starts and cDNA-ends at the start of Y-tracts. Figure S4. Constrained cDNA-ends in eIF4A3 iCLIP. Figure S5. The impact of cDNA-end constraints on cDNA-starts in eIF4A3 iCLIP. Figure S6. Distribution of cDNA sizes in the studied experiments. (PDF 5.42 mb) [file 13059_2016_1130_MOESM1_ESM.pdf]
